# Supplementary material for: Learning, sleep replay and consolidation of contextual fear memories: A neural network model
Source: PLoS Comput Biol. 2026 Mar 17;22(3):e1013251. doi: 10.1371/journal.pcbi.1013251 (PMC13012624; doi:10.1371/journal.pcbi.1013251)
Supplement: S1 Appendix — (PDF) [file pcbi.1013251.s001.pdf]

S1 Appendix Formal Model Description

As per Fig 1, each *module* of our model, such as HIP, CTX or BA_N, belongs to one of three kinds of network – Bayesian Confidence Propagation Network (BCPNN), k-Winner Takes All (kWTA) or 'Binary'. We adopted the auto-associative BCPNN architecture from Fiebig et al. [1]. A full discussion of the network architecture can be found there, or in Sandberg et al. [2]. Below, we provide a full formal description of our model. But first, we provide, as a summary, a broad description of the technical building blocks on which our implementation is based.

CTX, our model's 'long-term memory module', is a Bayesian Confidence Propagation Neural Network (BCPNN). This network archetype, developed by Lansner and colleagues [3], was shown capable of acting as an auto-associative ([4]) memory system [1, 2]. Its units are grouped into 'hypercolumns', within which they weakly compete for activity – a principle observed in the cerebral cortex [5]. The network is fully connected, with recurrent weights W enabling excitatory inputs, whose strength is modulated by a 'gain' or 'conductance' g_L. Weights are updated via a Hebb-like learning rule, derived from a Naive Bayes classifier [2], at a rate inverse to time constant τ_L. Optionally, to prevent the indefinite repetition of an attractor state in the absence of further input, complementary weights V, updated using a much lower (faster) time constant τ_A, may inhibit excitatory inputs, mediated by *negative* gain g_A. In the biological brain, a corresponding mechanism for discouraging neural patterns that have recently fired is observed, e.g., as the temporary depression of synapses between pyramidal cells recently involved in the same 'population burst' [6]. The updating equations defining a BCPNN are outlined below and discussed in greater detail by other sources [1, 2].

In a BCPNN, a softmax function is used on any time step to determine the neurons' activities within each hypercolumn. The fraction of strongly contributing units may vary considerably between different attractors. This feature is one reason we – like Fiebig & Lansner before us [1] – opt to use this architecture only for our model's cortical module. In subcortical regions of the biological brain that form engrams to encode sensory or contextual inputs, the size of these engrams is remarkably consistent (with a sparsity of, e.g. $2-6\%$ in dentate gyrus or $10-20\%$ in lateral amygdala [7]). To enforce such stable engram sizes, our model's 'engram layers' apart from CTX use a kWTA- rather than softmax rule to compute neurons' activities from their inputs – only the $K = \alpha N$ (sparsity times network size) units with the highest net input are active at any point in time. We thus distinguish between BCPNN- (CTX) and kWTA (HIP, BLA) modules. However, the Hebbian learning rules for updating the modules' weights (W and V)- apply to both kinds equally.

While this kWTA approach is suitable for storing a fixed-size engram – a 'discrete index' – for a given input, it does not allow the corresponding network to become inactive without further modulation. To associate a continuous quantity with a given engram, past models have assumed the needed information to be encoded in the (real-valued) strength of synapses extending from the engram cells [8–10]. Demonstrating an alternative mechanism, we allow the engrams of our model to recruit individual (e.g., fear-coding) cells out of a larger population to encode an associated quantity in the number of such neurons that are activated. For simplicity, we model such populations as sets of units without recurrent synapses. Any unit receives its input solely from synapses from other modules (see below). If the net input exceeds a chosen threshold, the neuron (which we then call 'recruited') fires (at a rate of 1).

Lastly, any two modules may be uni-directionally linked by a *Connection* instance. The input from source unit i to target unit j is computed by multiplying current activity $\pi_{source,i}$ with $g_{FB} \log(W_{ij})$, where 'feedback gain' g_{FB} is predetermined for each *Connection* and where the updating rules for weights W follow naturally from the

BCPNN architecture. Alternatively, a *Connection* may be non-plastic, in which case W is set or randomly generated when the model is initialized.

In the following, our architecture's building blocks are outlined in more detail.

BCPNN

Here we present the core updating equations of the BCPNN. The model operates in discrete time steps of length $dt = 10ms$ (though the model should not be interpreted as strictly operating on this timescale; rather, its dynamics qualitatively represent repetitive neural firing over longer durations). On each time step, the net excitatory input h received by each unit in a BCPNN evolves according to Equations (1) through (10) below.

The network is organized into hypercolumns H_k, each containing M units. The activity of units within a hypercolumn is normalized, simulating lateral inhibition mediated by inhibitory interneurons. This organization allows the network to operate as a soft-winner-takes-all (WTA) system. Groups of neurons that qualitatively operate in this manner have been described as the 'basic functional units' of neural computation in the cerebral cortex [5].

Dynamics of Net Input

The net input $h_j(t)$ received by unit j is updated based on the activity of other units in the network and the synaptic weights. Two types of connections contribute to this update:

- **Excitatory connections**, with weights $w_{ij}(t)$ and biases $\beta_i(t)$, which implement Hebbian learning.
- **Inhibitory connections**, with weights $v_{ij}(t)$ and biases $\gamma_i(t)$, which implement an adaptation mechanism that discourages prolonged firing of the same cell ensemble.

The net input evolves as follows:

$$\begin{aligned}\frac{dh_j(t)}{dt} = g_L &\left[\beta_j(t) + \sum_k \log\left(\frac{\sum_{i\in H_k} w_{ij}(t)\hat{\pi}_i(t)}{\hat{\pi}_j(t)}\right)\right] \\ &+ g_A\left[\gamma_j(t) + \sum_k \log\left(\frac{\sum_{i\in H_k} v_{ij}(t)\hat{\pi}_i(t)}{\hat{\pi}_j(t)}\right)\right] \\ &+ \text{Feedback Input}_j - h_j(t) \qquad (1)\end{aligned}$$

Here, Feedback Input_j refers to the summed input that unit j receives from *other* modules. Our implementation of such *inter-modular connections* is outlined further below.

Activity Normalization

The output activity $\hat{\pi}_j(t)$ of each unit is computed via a softmax function, modelling lateral inhibition within hypercolumns:

$$\hat{\pi}_j(t) = \frac{e^{h_j(t)}}{\sum_{j\in H(j)} e^{h_j(t)}} \qquad (2)$$

Hebbian Learning

Hebbian learning updates excitatory synaptic weights $w_{ij}(t)$ and biases $\beta_i(t)$ based on running averages of unit activities. A 'minimal background activity' parameter λ_0 ensures numerical stability and prevents extreme weight values in the absence of input.

$$\tau_L \frac{d\Lambda_i(t)}{dt} = \hat{\pi}_i(t) - \Lambda_i(t) \tag{3}$$

$$\tau_L \frac{d\Lambda_{ij}(t)}{dt} = \hat{\pi}_i(t)\hat{\pi}_j(t) - \Lambda_{ij}(t) \tag{4}$$

$$\beta_i(t) = \log(\Lambda_j(t)) \tag{5}$$

$$w_{ij}(t) = (1-\lambda_0^2)\Lambda_{ij}(t) + \lambda_0^2[(1-\lambda_0)\Lambda_i(t) + \lambda_0][(1-\lambda_0)\Lambda_j(t) + \lambda_0] \tag{6}$$

Adaptation Mechanism

An analogous process governs inhibitory weights $v_{ij}(t)$ and biases $\gamma_i(t)$, which are updated on a slower timescale (governed by τ_A):

$$\tau_A \frac{d\mu_i(t)}{dt} = \hat{\pi}_i(t) - \mu_i(t) \tag{7}$$

$$\tau_A \frac{d\mu_{ij}(t)}{dt} = \hat{\pi}_i(t)\hat{\pi}_j(t) - \mu_{ij}(t) \tag{8}$$

$$\gamma_i(t) = \log(\mu_j(t)) \tag{9}$$

$$v_{ij}(t) = (1-\lambda_0^2)\mu_{ij}(t) + \lambda_0^2[(1-\lambda_0)\mu_i(t) + \lambda_0][(1-\lambda_0)\mu_j(t) + \lambda_0] \tag{10}$$

kWTA Network

A k-Winner-Takes-All (kWTA) network is a variation on the BCPNN architecture described above. Unlike the BCPNN, the kWTA network does not organize its units into hypercolumns. Instead, the entire network competes globally, with only the k most active units at each time step producing non-zero activity. This approach, adopted from Fiebig & Lansner [1], more strictly enforces sparse network activity using a 'hard WTA' constraint.

On each time step, every unit computes its net excitatory input h, which evolves as follows:

$$\begin{aligned}\frac{dh_j(t)}{dt} = g_L &\left[\beta_j(t) + \log\left(\sum_i w_{ij}(t)\pi_i(t)\right)\right] \\ &+ g_A\left[\gamma_j(t) + \log\left(\sum_i v_{ij}(t)\pi_i(t)\right)\right] \\ &+ \text{Feedback Input}_j - h_j(t)\end{aligned} \tag{1}$$

Here, $w_{ij}(t)$ and $\beta_j(t)$ represent the excitatory synaptic weights and biases, while $v_{ij}(t)$ and $\gamma_j(t)$ represent the inhibitory weights and biases. As previously, the terms g_L and g_A are scaling factors controlling the relative contributions of excitatory and inhibitory inputs.

Activity Update: Hard WTA Rule

Unit activities in a kWTA network are binary (0 or 1) and determined using a hard WTA rule. At each time step, the k units with the highest $h_j(t)$ values are assigned an

activity of 1, while the rest are set to 0:

$$\pi_j(t) = \begin{cases} 1, & h_j(t) \text{ is in the top } k \text{ values} \\ 0, & \text{otherwise.} \end{cases} \tag{12}$$

Here, k is given as the product of the total number of units (N) and a sparsity parameter (α). The kWTA network implements Hebbian learning and an adaptation mechanism using the same updating equations (3) to (10) as described for the BCPNN.

Binary Module

Both BCPNNs and kWTA Networks effectively serve the purpose of auto-associatively storing and remembering their own activity patterns. This key feature makes them suitable for the CTX, HIP and $\mathrm{BA_N}$ modules of our model. In contrast, our model's $\mathrm{BA_P}$ and $\mathrm{BA_I}$ modules can be conceptualized as populations of – non-interacting, for simplicity – cells that can be *recruited* by certain activity patterns of *other* modules. If a context is assumed to be encoded by a fixed activity pattern in $\mathrm{BA_N}$, then *associating valence* with that context is equivalent to *strengthening plastic synapses* from that $\mathrm{BA_N}$ ensemble to a selection of units in $\mathrm{BA_P}$ and/or $\mathrm{BA_I}$. $\mathrm{BA_P}$ and $\mathrm{BA_I}$ are implemented as 'Binary Modules'.

The Binary Module operates by comparing the net input $h_j(t)$ received by each unit j to a fixed firing threshold θ. Units are activated (assigned a binary activity of 1) if their net input exceeds this threshold. The dynamics of net input and activity update are given by:

$$\frac{dh_j(t)}{dt} = \text{Feedback Input}_j - h_j(t) \tag{13}$$

$$\pi_j(t) = \begin{cases} 1, & h_j(t) > \theta \\ 0, & \text{otherwise.} \end{cases} \tag{14}$$

Hence, units of these modules receive their input entirely from other modules. This architecture enables $\mathrm{BA_P}$ and $\mathrm{BA_I}$ to encode valence-related quantities by recruiting varying numbers of active units.

Inter-Modular Connections

In the sections above, we wrote Feedback Input_j to refer to the net input received by unit j from modules other than the one to which that unit belongs. The transmission of inputs from a *source* to a *target* module works very similarly to the transmission of excitatory inputs within a BCPNN or kWTA module.

Updating Feedback Inputs

The feedback input to a target unit j is computed as a weighted sum of the activity of units in the source module, normalized and passed through a logarithmic activation. For each target unit j, the feedback input is given by:

$$\text{Feedback Input}_j = g_{\text{FB}} \log \left(\sum_i W_{ij} \pi_i^{\text{source}}(t) \right) \tag{15}$$

Here, W_{ij} represents the synaptic weight between source unit i and target unit j, and $\pi_i^{\text{source}}(t)$ is the output activity of source unit i at time t. The term g_{FB} is a gain parameter controlling the contribution of the feedback input.

If inter-modular connections from multiple *source* modules are defined for a single *target* module, the contributions of these connections are summed.

Hebbian Learning for Inter-modular Weights

Inter-modular connection weights W_{ij} adapt according to a Hebbian learning rule, driven by joint activity estimates between the source and target units:

$$\tau_{\text{fb}} \frac{d\Lambda_{ij}(t)}{dt} = \pi_i^{\text{source}}(t)\pi_j^{\text{target}}(t) - \Lambda_{ij}(t) \tag{16}$$

Here, $\Lambda_{ij}(t)$ is a running average that estimates the joint activity of source unit i and target unit j. These estimates are updated continuously over time with a timescale governed by τ_{fb}.

The weights $W_{ij}(t)$ are computed from these activity estimates:

$$W_{ij}(t) = \frac{(1-\lambda_0^2)\Lambda_{ij}(t) + \lambda_0^2}{\left[(1-\lambda_0)\Lambda_i^{\text{source}}(t) + \lambda_0\right]\left[(1-\lambda_0)\Lambda_j^{\text{target}}(t) + \lambda_0\right]} \tag{17}$$

Here, $\Lambda_i^{\text{source}}(t)$ and $\Lambda_j^{\text{target}}(t)$ are marginal activity estimates of the source and target units, respectively – which are computed by the source and target *modules*, as per equation (3). As previously, the background activity parameter λ_0 ensures numerical stability and prevents weights from diverging when activity levels are very low.

Model parameters

Module Name	Module Type	No. Units	Comment
SC	-	500	Activity manually set.
EC_{IN}	Binary	500	cf. S1 Fig.
HIP	kWTA	350	
EC_{OUT}	Binary	500	cf. S1 Fig.
CTX	BCPNN	500	10 units per hypercolumn.
$\mathrm{BA_N}$	kWTA	500	
$\mathrm{BA_P}$	Binary	250	
$\mathrm{BA_I}$	Binary	250	

Table A. List of all modules in the model, their type, and number of units. The Sensory Cortex (SC) is special in that its activity is always manually set to a pattern encoding the current environment – or silenced during *Sleep*. Hence it does not actually behave like a BCPNN and effectively has no internal weights.

Source Module	Target Module
HIP	EC_{OUT}
HIP	CTX
CTX	$\mathrm{BA_N}$
HIP	$\mathrm{BA_N}$
$\mathrm{BA_N}$	$\mathrm{BA_P}$
$\mathrm{BA_N}$	$\mathrm{BA_I}$

Table B. List of all plastic inter-modular connections in the model, as per Fig 1.

Module	Parameter symbol	Perception	Sleep	Recall
**Module Parameters**				
EC_{IN}	θ	0.75	0.75	0.75
HIP	τ_L	350	∞	∞
	g_L	0.0	1.0	1.0
	τ_A	∞	1200	∞
	g_A	0.0	-0.85	0.0
	α (sparsity)	0.04	0.04	0.04
EC_{OUT}	θ	0.75	2.75	2.75
CTX	τ_L	36,000	18,000	∞
	g_L	0.0	0.0	1.0
$\mathrm{BA_N}$	g_L	0.0	0.0	0.05
	τ_L^{slow}	72,000	∞	∞
	τ_L^{fast}	400	∞	∞
	α (sparsity)	0.10	0.10	0.10
$\mathrm{BA_P}$	θ	3.05	3.05	3.05
$\mathrm{BA_I}$	θ	4.50	4.50	4.50
**Connection Parameters**				
$HIP \rightarrow EC_{OUT}$	g_{FB}	0.0	1.0	1.0
	τ_{FB}	800	∞	∞
$HIP \rightarrow CTX$	g_{FB}	0.0	1.0	0.0
	τ_{FB}	100	∞	∞
$HIP \rightarrow BA_N$	g_{FB}	0.0	1.0	0.0 / 1.0
	τ_{FB}^{fast}	150	∞	∞
	τ_{FB}^{slow}	10,000	∞	∞
$CTX \rightarrow BA_N$	g_{FB}	0.0	0.0	0.0 / 1.0
	τ_{FB}^{fast}	10,000	20,000	∞
	τ_{FB}^{slow}	30,000	20,000	∞
$BA_N \rightarrow BA_P$	g_{FB}	1.0	1.0	1.0
	τ_{FB}^{fast}	1,000	∞	∞
	τ_{FB}^{slow}	∞	∞	∞
$BA_N \rightarrow BA_I$	g_{FB}	1.0	1.0	1.0
	τ_{FB}^{fast}	2,000	∞	∞
	τ_{FB}^{slow}	∞	∞	∞

Table C. Module and connection parameters during Perception, Sleep, and Recall. θ denotes a binary module's firing threshold. τ_L, τ_A, and τ_{FB} are learning time constants for recurrent excitatory, inhibitory, and inter-modular weights, respectively; g_L, g_A, and g_{FB} are the corresponding gains.
During *Recall*, *either* the gain g_{FB} of the $HIP \rightarrow BA_N$ connection *or* of the $CTX \rightarrow BA_N$ connection equals 1.0 – and the other equals 0.0. This depends on whether HIP successfully retrieved a memory (cf. S1 Fig) – when this is not the case, 'control' over $\mathrm{BA_N}$ defaults to CTX.

Name	Description	Value
λ_0	'Background activity' constant, placing lower and upper bounds on synapse strengths (see above).	0.025
B_{AMY}	If the F1 score between EC_{in} and EC_{out} activities exceeds this threshold, the activity in HIP appears to match the current sensory input during *Recall* (cf. S1 Fig); then, HIP is in control of BA_N. Else, the gain of $HIP \to BA_N$ is set to 0 and that of $CTX \to BA_N$ to 1.	0.55
r_{homeo}	Rate of change of the homeostasis rule acting on $BA_N \to BA_P$ and $BA_N \to BA_I$ synapses	0.01
T_{stress}	If π_A (a running average of recent US delivery, cf. S2 Appendix Stage 3) exceeds this threshold, it triggers the model's 'extreme stress' effects (SEFL).	0.9
$T^P_{\mathrm{ext,default}}$	Default pruning threshold for $\mathrm{BA_N} \to \mathrm{BA_P}$ homeostasis rule.	0.175
$T^P_{\mathrm{ext,min}}$	Minimum pruning threshold for $\mathrm{BA_N} \to \mathrm{BA_P}$ homeostasis rule (activated by 'extreme stress').	0.05
$T^{P,\mathrm{recovery}}_{\mathrm{ext}}$	Recovery rate of pruning threshold after 'extreme stress', per time step.	1/6,000
recruitment_level_P	Target strength of $\mathrm{BA_N} \to \mathrm{BA_P}$ synapses *consolidated* by homeostasis rule (if their strength initially exceeds $T^P_{\mathrm{ext,min}}$)	0.45
$q_{\mathrm{P,default}}$	Default normalization divisor for P-cell recruitability (cf. S2 Appendix).	2.0
$q_{\mathrm{P,min}}$	Minimum normalization divisor for P-cell recruitability (activated by 'extreme stress').	1.75
$q^{\mathrm{recovery}}_{\mathrm{P}}$	Recovery rate of P-cell recruitability normalization divisor.	1/12,000
$T^I_{\mathrm{ext,default}}$	Pruning threshold for $\mathrm{BA_N} \to \mathrm{BA_I}$ homeostasis rule.	0.4
recruitment_level_I	Target strength of $\mathrm{BA_N} \to \mathrm{BA_I}$ synapses *consolidated* by homeostasis rule	0.9
q_{I}	Normalization factor for I-cell recruitability (cf. S2 Appendix).	0.5
$p_{\mathrm{ff}\to c}$	Feedforward gain from $\mathrm{BA_P}$ to CeA	1.0
$i_{\mathrm{ff}\to c}$	Feedforward gain from $\mathrm{BA_I}$ to CeA	-1.0
$u_{\mathrm{ff}\to p}$	'Increment for plasticity' from (US signal) U to $\mathrm{BA_P}$ (cf. S2 Appendix, Stage 2, Step 9)	3.0
$c_{\mathrm{ff}\to p}$	'Decrement for plasticity' from CeA to $\mathrm{BA_P}$	-3.0
$u_{\mathrm{ff}\to i}$	'Decrement for plasticity' from U to $\mathrm{BA_I}$	-4.25
$c_{\mathrm{ff}\to i}$	'Increment for plasticity' from CeA to $\mathrm{BA_I}$	4.25

Table D. Remaining model parameters. These are parameters of the model that do not belong to any specific module or plastic connection, which are listed in Table C.

References

1. Fiebig F, Lansner A. Memory consolidation from seconds to weeks: a three-stage neural network model with autonomous reinstatement dynamics. Frontiers in computational neuroscience. 2014;8:64.

2. Sandberg A, Lansner A, Petersson KM, Ekeberg. A Bayesian attractor network with incremental learning. Network: Computation in neural systems. 2002;13(2):179-94.

3. Lansner A, Ekeberg Ö. A one-layer feedback artificial neural network with a Bayesian learning rule. International journal of neural systems. 1989;1(01):77-87.

4. Treves A, Rolls ET. What determines the capacity of autoassociative memories in the brain? Network: Computation in Neural Systems. 1991;2(4):371.

5. Molnár Z, Rockland KS. Cortical columns. In: Neural circuit and cognitive development. Elsevier; 2020. p. 103-26.

6. Tsodyks M, Uziel A, Markram H. Synchrony generation in recurrent networks with frequency-dependent synapses. The Journal of neuroscience. 2000;20(1):RC50.

7. Rao-Ruiz P, Yu J, Kushner SA, Josselyn SA. Neuronal competition: microcircuit mechanisms define the sparsity of the engram. Current opinion in neurobiology. 2019;54:163-70.

8. Krasne FB, Cushman JD, Fanselow MS. A Bayesian context fear learning algorithm/automaton. Frontiers in behavioral neuroscience. 2015;9:112.

9. Krasne FB, Zinn R, Vissel B, Fanselow MS. Extinction and discrimination in a Bayesian model of context fear conditioning (BaconX). Hippocampus. 2021;31(7):790-814.

10. Krasne FB, Fanselow MS. Remote memory in a Bayesian model of context fear conditioning (BaconREM). Frontiers in Behavioral Neuroscience. 2024;17:1295969.
