# Supplementary material for: Learning, sleep replay and consolidation of contextual fear memories: A neural network model
Source: PLoS Comput Biol. 2026 Mar 17;22(3):e1013251. doi: 10.1371/journal.pcbi.1013251 (PMC13012624; doi:10.1371/journal.pcbi.1013251)
Supplement: S2 Appendix — (PDF) [file pcbi.1013251.s002.pdf]

S2 Appendix Full Update Cycle of the Model

This section describes the step-by-step update process of the model during a single simulation cycle. It outlines the sequence in which modules and connections are updated, to provide a clearer picture of how the model operates. The underlying Python code is provided, as per the data availability statement in the main text.

Stage 1 – Updating the Context-encoding Modules(HIP, CTX, BA_N)

If the model currently receives an environmental input (`sensory_input` – an abstract, binary vector of length 500) – the activity of the engram modules is updated based on this input and pre-defined connection weight matrices. These feedforward connections are constant over time and are initialized as follows: $W_{SC \to EC_{IN}}$ is set to the identity matrix I, $W_{SC \to CTX} = 8I$, whereas $W_{SC \to BA\_N}$ is a random permutation of the identity matrix. Entries of $W_{EC_{IN} \to HIP}$ are drawn uniformly at random from $[0, 1]$.

The following sequence occurs:

1. **Sensory Cortex Activity**: The output of the Sensory Cortex module (SC), π_{SC}, is manually set to the provided `sensory_input`.
2. EC_{IN} **Activity**: Sensory Cortex activity is forwarded to EC_{IN}. Each unit in EC_{IN} receives a binary input given by $(W_{SC \to EC_{IN}})(\pi_{SC})$ and attains it as its output to propagate the signal to HIP.
3. **Hippocampus (HIP) Activity**: The net input received by any unit in HIP is given by $(W_{EC\_in \to HIP})(\pi_{EC_{IN}})$. The $\text{sparsity} * \text{No. units} = \alpha * N = 0.04 * 350 = 14$ most strongly innervated units become active, as per equation (12). Note that previously strengthened, recurrent synapses in HIP play no role at this point, as HIP activity during *memory formation* should be driven by external inputs rather than existing memories [1].
4. EC_{OUT} **Activity**: If the model is in **Perception** mode, the activity pattern of EC_{IN} is copied over to EC_{OUT} (cf. S1 Fig).
5. **Cortex (CTX) and Amygdala ($\mathbf{BA_N}$) Activity** : Similar to step 3, the units of CTX and BA_N receive their input via $(W_{SC \to CTX})(\pi_{SC})$ or $(W_{SC \to BA_N})(\pi_{SC})$ and attain their output as per equations (2) or (12), respectively.
6. **Within-Module Learning**: Now that the output of HIP, CTX and BA_N reflects the current environmental input, recurrent weights and related parameters of the modules are updated to support storing these activity patterns – unless the corresponding learning time constant τ_L is set to ∞. These updates occur via equations (3) to (10) above.

If, on the other hand, no environmental input is present (say, during *Sleep*), the sequence of steps is shorter.

1. Update HIP, EC_{OUT} and CTX, in that order, as described by equations (1) to (12).
2. **Assessing validity of HIP Activity**: If the model is in **Recall** mode, an F1 Score is computed between the binary activity vectors of EC_{IN} and EC_{OUT}. This measures whether HIP has *recalled* an engram that, during memory formation, was tied to an EC_{OUT} pattern that matches the current EC_{IN} activity. When this F1 Score exceeds 0.55, we say that HIP has successfully recalled a memory matching the current context. Then, gain g_{FB} of the $HIP \to BA_N$ connection is set to 1.0 and that of the $CTX \to BA_N$ connection is set to 0.0. Otherwise, the opposite occurs (cf. S1 Fig).

3. Update $\mathrm{BA_N}$, as described by equations (1) to (10).

In summary, in stage 1 of the update cycle, environmental inputs are passed to the context-encoding modules HIP, CTX and $\mathrm{BA_N}$. Learning rate parameters permitting, the resulting activity patterns are stored via Hebbian learning for future re-activation. When no environmental inputs are present (*Sleep* or *Recall*), the modules' activities are determined by previously learned recurrent weights; new learning may yet occur during *Sleep*, as per Table C in S1 Appendix. Note that, in *Perception* mode, the model always receives an environmental input.

Stage 2 – Activating a Fear Response and Inter-modular Plasticity

Once a representation of the current – or currently recalled – context has been established in HIP, CTX and $\mathrm{BA_N}$, the model must activate a fear response and/or change the amount of fear associated with that context, as appropriate.

This occurs as follows:

1. The *recruitability* of all units in $\mathrm{BA_P}$ and $\mathrm{BA_I}$ is updated – according to a procedure which, for brevity, is described in a subsequent section of the appendix.

2. The activity of the model's fear-evoking and -inhibiting cells, $\mathrm{BA_P}$ and $\mathrm{BA_I}$, is determined depending on plastic inputs arriving from $\mathrm{BA_N}$, as per equations (13) and (14).

3. CeA – the single unit encoding the model's current fear response – receives a net input h_{CeA} equal to the number of active units in $\mathrm{BA_P}$, minus the number of active units in $\mathrm{BA_I}$. Its output, π_{CeA} is set to

$$\frac{1}{1 + \exp(-0.3 * (h_{CeA} - 8.0))}.$$

A sigmoid function is applied to CeA's input to ensure that its output lies between 0 and 1 – the range of US strengths in our simulations.

4. The activity of the model's *U-cell*, π_U is set to the magnitude of the US signal currently delivered by us, the experimenter. Whenever no US is delivered, π_U remains 0.

5. The activity of the model's *A-cell*, intended to serve as a measure of *acute stress*, is updated as follows:

$$\pi_A \leftarrow \begin{cases} \pi_A + 0.4(\pi_U - \pi_A) & \text{if } \pi_U > \pi_A, \\ \pi_A + 0.05(\pi_U - \pi_A) & \text{otherwise.} \end{cases}$$

In words, the stress level (π_A) adjusts towards the US level (π_U). Stress is assumed to rise more quickly when a US newly occurs or is strengthened than it falls when a US ceases or is weakened.

6. The difference $\Delta = \pi_{CeA} - \pi_U$ is recorded. We interpret it as a signed prediction error for the strength of the currently observed US.

7. If the absolute value of the prediction error, $|\Delta|$, exceeds 0.2, the learning time constants τ_L (or τ_{FB}) of the recurrent $\mathrm{BA_N}$ synapses and of the plastic $HIP \rightarrow BA_N$ and $CTX \rightarrow BA_N$ connections are set to their *fast* values ($\tau^{fast}_{L/FB}$ in Table **??**). Else, the *slow* values are chosen.

8. If $\Delta < -0.05$, i.e. if $\pi_U > \pi_{CeA} + 0.05$, the learning time constant of the $BA_N \rightarrow BA_P$ connection is set to its *fast* value, τ_{FB}^{fast}, *divided* by $|\Delta|$. This allows fear-evoking units in BA_P to be recruited – and more quickly so when a *very* unexpected US occurs. The learning time constant of $BA_N \rightarrow BA_I$ is set to its *slow* value.

   Vice versa, if $\Delta > 0.05$, the learning time constant of $BA_N \rightarrow BA_I$ is set to τ_{FB}^{fast} divided by Δ whereas that of $BA_N \rightarrow BA_P$ is set to τ_{FB}^{slow} (cf. S1 Appendix).

9. Now that the outputs of all of the model's units have been set, learning takes place for all inter-modular, plastic synapses (cf. Table B in S1 Appendix), as per equations (16) and (17).

   An important particularity is necessary to allow the ('Non-Hebbian') recruitment of valence-coding units that are not yet active. When updating the $BA_N \rightarrow BA_P$ and $BA_N \rightarrow BA_I$ connections, equation (16) uses the activity $\pi_j^{target}(t)$ that the respective P- or I-cell *would have* if its net input were increased by the term

   $$3.0(\pi_U - \pi_C) \text{ for P-cells}$$

   or

   $$4.25(\pi_C - \pi_U) \text{ for I-cells,}$$

   multiplied by that unit's current *recruitability* value.

Stage 3 – Sleep Homeostasis

The third and final part of the model's update cycle consists of its sleep homeostasis mechanism – which we motivated and described in the main text of this paper.

The following steps are carried out:

1. If the *A-cell* activity, π_A lies above $T_{stress} = 0.9$, the *extinction threshold*, A_P, of the homeostasis rule acting on BA_P is lowered from its default value 0.175 to 0.05 (favouring the long-term recruitment of fear-evoking units, as outlined below). The value of T_{stress} was chosen so that this putative stress effect only occurs after the delivery of extremely strong USs over several time consecutive time steps – such as in the experimental SEFL paradigm [2].

2. If the value of A_P currently lies below its default value of 0.175, it is incremented by $\frac{1}{6,000}$. A full recovery of A_P thus occurs after $(0.175 - 0.05) * 6,000 = 750$ time steps – corresponding to roughly 3 full day-night cycles in our simulations. Stress-induced changes to the recruitability of BA_P cells are described further below.

If the model is not currently in *Sleep* mode, the update cycle ends at this point. Otherwise, homeostatic changes are applied to synapses onto valence, coding amygdala cells, as follows:

1. Define W_P to be the weight matrix of the plastic $BA_N \rightarrow BA_P$ connection, restricted to those rows that correspond to units in BA_N that are currently active.

2. Update the weights in W_P as per the cubic homeostasis rule

   $$\frac{dW_P}{dt} = r_P * W_P * (1 - \frac{W_P}{K_P}) * (\frac{W_P}{A_P} - 1),$$

where A_P is as described above, and where $r_P = 0.01, K_P = 0.45$. Synaptic weights that would fall below 0 are set to 0 instead. The larger the gap between A_P and K_P, the more stable the recruitment of P-cells by context-coding coding $\mathrm{BA_N}$ units.

3. To ensure that future updates to these synaptic weights will correctly account for these homeostatic changes (cf. Equation (16) above), the values of the 'running average' Λ_{ij} must be updated by inverting equation (17), as follows:

$$\Lambda_{ij}(t) = \frac{W_{ij}(t)\left[(1-\lambda_0)\Lambda_i^{\text{source}}(t) + \lambda_0\right]\left[(1-\lambda_0)\Lambda_j^{\text{target}}(t) + \lambda_0\right] - \lambda_0^2}{1-\lambda_0^2}. \quad (1)$$

The meaning of the involved symbols is as described for equation (17).

4. Repeat the above steps 1 to 3, but for $\mathrm{BA_I}$ rather than $\mathrm{BA_P}$, with $r_I = 0.01, A_I = 0.4, K_I = 0.9$.

Recruitability of P- and I-cells

As presaged in Stage 2, Step 1 of the above update cycle, here we provide a formal definition of the way that the 'recruitability' of valence-coding amygdala ($\mathrm{BA_P}$ and ($\mathrm{BA_I}$) cells evolves in our model. As per Stage 2, Step 9, the current recruitability of each cell influences the likelihood of it being recruited into a context representation in case of a conditioning event. Computationally, the point of including such a parameter was to limit the amount of fear/extinction generalisation in the model – if conditioning in different environments always involved the same valence-coding cells, plasticity on the relevant synapses would quickly saturate.

The concept of a temporally evolving, cell-intrinsic *bias* affecting the allocation of neurons to newly formed activity patterns has previously been studied in the biological brain [3,4], as well as in silico [5]. Yet, there is no clear consensus on how such a bias is likely to 'behave'. We thus had a lot of freedom in our implementation, but formulated a list of design targets to guide us:

1. Each cell alternates between periods of high and low recruitability.

2. At any given time, only a relatively small fraction of valence-coding cells should be 'highly recruitable', to prevent a saturation of synapses (cf. above).

3. Groups of valence-coding cells that are simultaneously highly recruitable are broken up over time, so that – eventually – any combination of valence-coding cells may potentially appear in an engram. This is highly putative but, e.g., counteracts fear sensitization that would occur if the exact same group of P-cells was recruited in two contexts.

4. High-recruitability periods last approximately 200 time steps. This time window is longer than most conditioning sessions, but shorter than a day-night cycle, in our simulations. Valence-coding cell ensembles recruited into different context engrams on the same day are thus likely to overlap, such as to *link* the resulting associative memories [5].

5. The total recruitability of the system remains relatively stable over time.

6. Recruitability is a continuous quantity, with the full range of possible values being present in the population at any point in time. This, e.g., allows the recruitment of *few* cells in case of significant-but-weak predictions.

Formally, the recruitability of the fear-evoking P-cells, of which there are 250, evolves as follows:

1. When the model is first initialized, a small subset of P-cells is selected, independently at random, using a binomial distribution with $p = 0.05$. Each selected P-cell receives a 'starting phase' θ, again independently at random, uniformly from the interval $[0, \pi]$. For each remaining P-cell, θ is drawn uniformly from $[-\pi, 0]$. Then, each P-cell receives its initial recruitability, computed as

$$\frac{1 + \sin(\theta)}{q_p} + \epsilon,$$

   where the ϵ-term denotes random noise, drawn from $\mathcal{N}(\mu = 0, \sigma = 0.25)$. The normalization divisor q_p is 2 by default, but can decrease under stress (see below). Negative recruitability values are set to 0.

2. On any subsequent time step, the *phase* of each P-cell whose *current* recruitability exceeds 0.5 is advanced by $\frac{\pi}{100}$. The duration for which cells remain *highly* recruitable is thus tightly regulated. On the other hand, the phase of each *remaining* P-cell has a (1/16)-chance of being advanced by $\frac{\pi}{25}$. The duration for which cells are *unrecruitable* or 'passive' thus varies randomly – serving design target 3.) above. Recruitability values are again computed as $\frac{1+\sin(\theta)}{q_P} + \epsilon$.
   Note: When the model enters its *stress mode* (see the first step in Stage 3), the divisor in this equation is decreased from 2 to 1.75, and then recovers linearly at a rate of 1/12000 per time step.

The recruitability of the fear-inhibiting I-cells behaves independently from P-cells, with a fixed normalization divisor of $q_I = 2$ and more slowly, with a phase advance of $\frac{\pi}{400}$ and 'random push' strength of $\frac{\pi}{100}$. See S2 Fig for a visualization of the rule here discussed.

References

1. Treves A, Rolls ET. Computational constraints suggest the need for two distinct input systems to the hippocampal CA3 network. Hippocampus. 1992;2(2):189-99.

2. Hassien AM, Shue F, Bernier BE, Drew MR. A mouse model of stress-enhanced fear learning demonstrates extinction-sensitive and extinction-resistant effects of footshock stress. Behavioural brain research. 2020;379:112391.

3. Park S, Kramer EE, Mercaldo V, Rashid AJ, Insel N, Frankland PW, et al. Neuronal allocation to a hippocampal engram. Neuropsychopharmacology. 2016;41(13):2987-93.

4. Choucry A, Nomoto M, Inokuchi K. Engram mechanisms of memory linking and identity. Nature Reviews Neuroscience. 2024:1-18.

5. Delamare G, Tomé DF, Clopath C. Intrinsic neural excitability biases allocation and overlap of memory engrams. Journal of Neuroscience. 2024;44(21).
