# Supplementary material for: Learning, sleep replay and consolidation of contextual fear memories: A neural network model
Source: PLoS Comput Biol. 2026 Mar 17;22(3):e1013251. doi: 10.1371/journal.pcbi.1013251 (PMC13012624; doi:10.1371/journal.pcbi.1013251)
Supplement: S3 Appendix — (PDF) [file pcbi.1013251.s003.pdf]

S3 Appendix Stability Analysis

Overview

The simulation results reported in this paper are intended to highlight our model's behaviour with regard to fear acquisition, extinction, generalization and renewal, as well as the our assumptions on the importance of sleep (homeostasis) for regulating synaptic density and preventing the emergence of a fear-sensitized state. To make clear which behaviours are robust within our model architecture, and which rely on more delicate parameter combinations, here we performed a series of stability analyses. Our general approach was the following:

In each of the following sections, we evaluated the robustness of a specific, qualitative simulation result. We did so by formalizing *under what conditions* that result can be said to hold. We then randomly re-sampled certain parameter choices, repeatedly subjected the altered models to the simulation protocol in question, and assessed the outcomes. Our model contains a large number of interacting parameters, making a systematic grid search infeasible. Hence, in each section, we varied only a subset of parameters – those we expected would be most influential for the result in question – while all others remained at their default values.

In each section, parameters were varied using Latin Hypercube Sampling (LHS) [1]. Several simulation runs were performed for each parameter combination, using a different *noise seed* each time, and averaging the relevant simulation outcomes across them.

Context specificity of fear extinction

Fig 4b of our main results demonstrates that, after performing fear acquisition and extinction in the same context A, some amount of fear will return when the model is exposed to a *moderately similar* context B. That is because the recruitment of BA_I cells into context representation is less stable than that of BA_P cells, resulting in a steeper generalization gradient.

We varied the following parameters, controlling the recruitment and activity of valence-coding BA_P and BA_I cells:

- θ_P (BA_P firing threshold): uniform in $[2.75, 3.5]$,
- $\Delta := \theta_I - \theta_P$: uniform in $[0.25, 0.75]$,
- $\tau_{\text{FB}}^{\text{fast}}$of $BA_N \to BA_I$ synapses ($\propto$ learning rate after positive prediction errors (US delivery)): log-uniform in $[500, 1500]$,
- $\tau_{\text{FB}}^{\text{fast}}$of $BA_N \to BA_I$ synapses ($\propto$ learning rate after negative prediction errors (US omission)): log-uniform in $[1500, 3000]$.

Each simulation run, with the same protocol as in Fig 5b), was repeated for 10 independent noise seeds. Averaging across those runs yielded a *generalization curve* $f(s)$ for each parameter setting – the mean CeA output in renewal contexts of different similarity s (to acquisition context A). We explored 600 parameter settings in total.

A parameter setting satisfied the **pass/fail criterion** if it met the following conditions:

- **Interior peak:** the maximum of $f(s)$ occurs at a non-endpoint similarity in the interval $[0.33, 0.975]$.
- **Renewal is low for dissimilar contexts:** the Spearman correlation between s and $f(s)$ on the points up to and including the peak exceeds 0.4.

- **Renewal is low for very similar contexts:** the Spearman correlation on the points from the peak to the end is below -0.4.
- **Fear expression is low at the boundaries of the curve:** Both $f(0)$ and $f(1)$ are not much greater than the lowest point of the curve. Formally, $f(s)$ has a 'peakedness index' of at least 0.25,

where we defined a 'peakedness index' as

$$PI = \frac{\max_s f(s) - \max\{f(0), f(1)\}}{\max_s f(s) - \min_s f(s)}.$$

A PI below 0.25 indicates that f attains a clear minimum *between* $s = 0$ and $s = 1$, meaning that f does not match the shape we report in our main results.

The overall shape of the generalization curve is quite robust. 540 out of the 600 samples passed our test (90.0%). Fig A shows the average curve across all *feasible* parameter sets – where we define a sample to be 'feasible' if extinction was successful (a decrease of the fear response of at least 0.5 at the end of extinction in A, compared to the end of the acquisition phase). Fig B, which shows the average curve across all samples that passed our criteria, is similar.

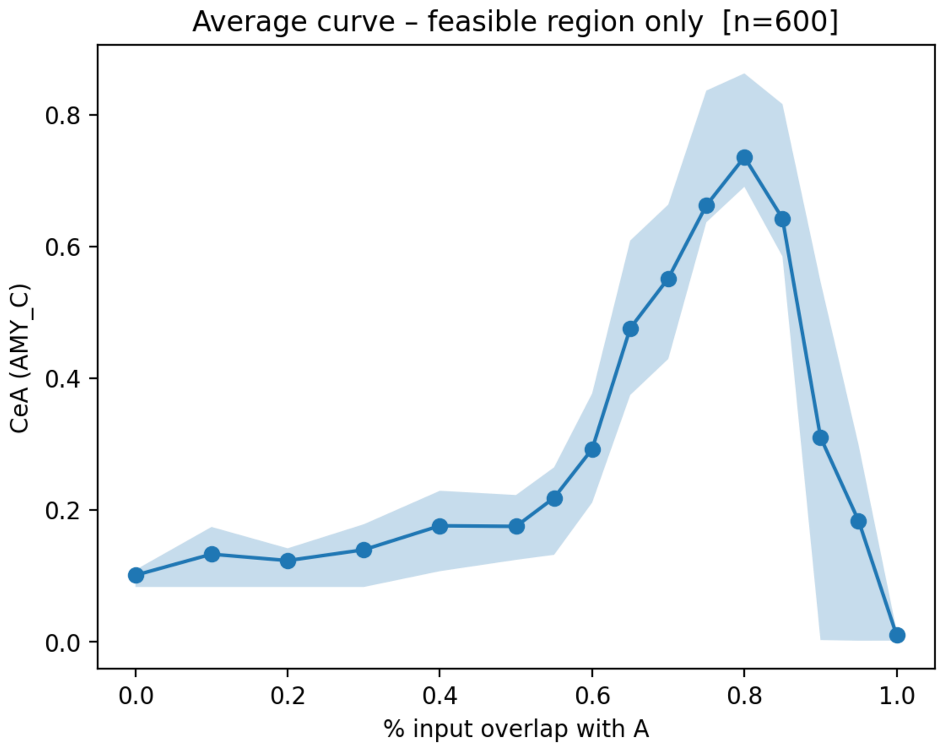

Fig A. Stability Analysis – Generalization gradient: Average generalization curve (all *feasible* samples). Mean generalization curve across all *feasible* parameter sets for which fear extinction was successful ($n = 600$ out of 600). Shaded bands show the interquartile range. The curve rises from low similarity, peaks at intermediate overlap, and then declines toward high similarity—consistent with the pattern reported in the main text.

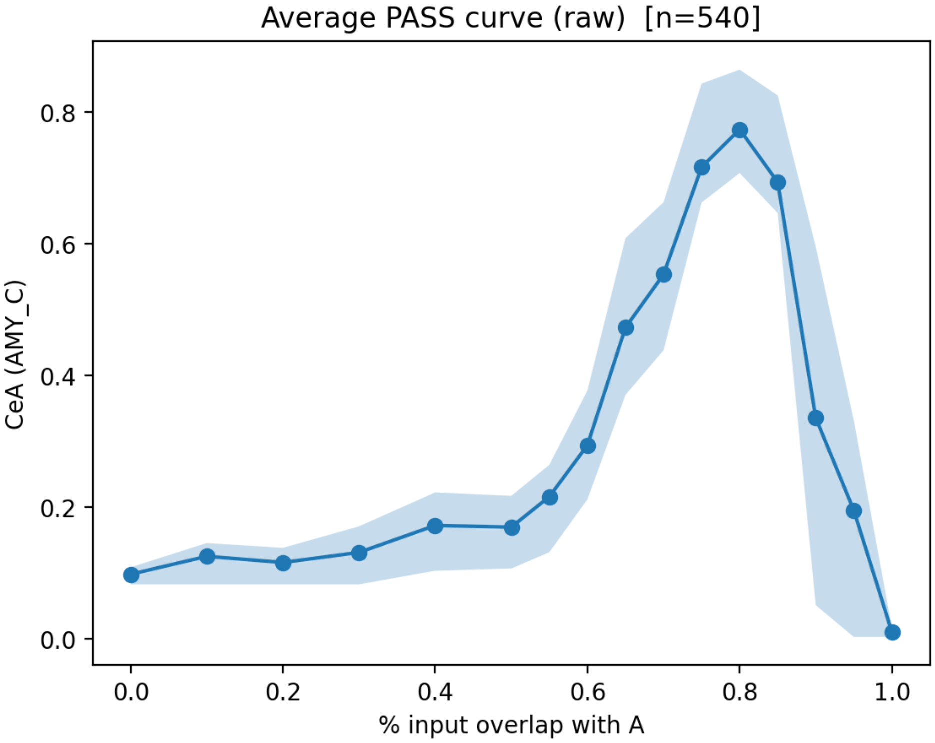

Fig B. Stability Analysis – Generalization gradient: Average generalization curve (PASS only). Mean generalization curve across all parameter sets that passed all four test criteria ($n = 540$ out of 600). Shaded bands show the interquartile range. The curve is similar to the curve across *all* parameter sets, but has a slightly sharper peak around a similarity of $s \simeq 0.7 - 0.8$.

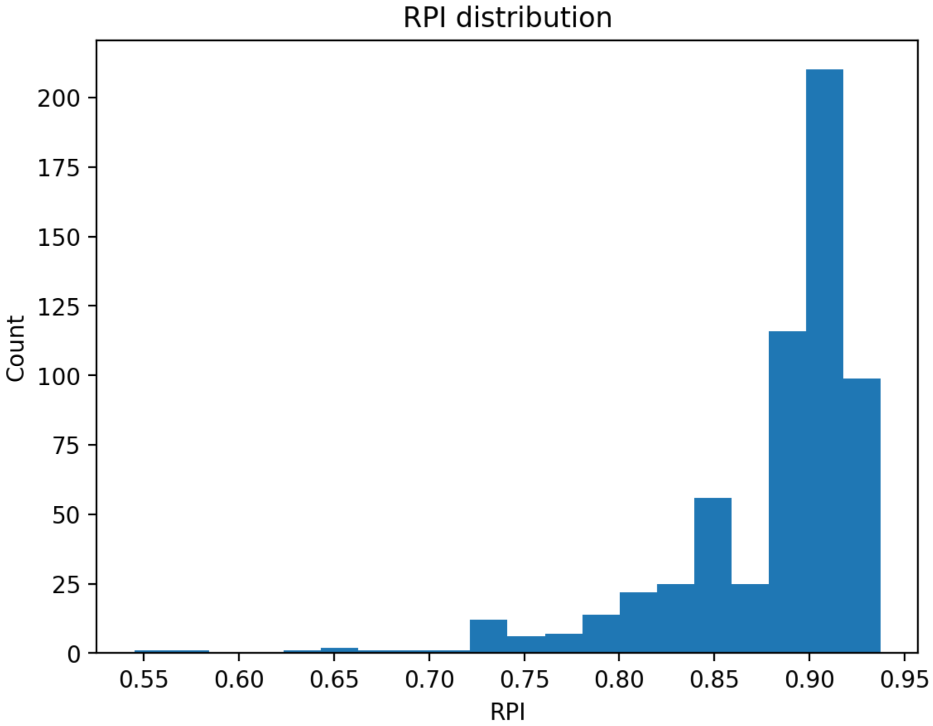

Fig C. Stability Analysis – Generalization gradient: Peakedness distribution. Histogram of the peakedness index (PI), as defined above, across all $n = 600$ samples. The distribution is skewed towards values near 1, indicating that a majority of assessed generalization curves $f(s)$ reached a clear peak at a similarity *within* $s \in (0, 1)$.

Increasing fear generalization with memory age

Fig 4d of our main results shows that, after fear is acquired in context A, the amount of fear that is expressed in a moderately similar, unconditioned context B increases over time as the fear memory ages. Meanwhile, fear expression in an unconditioned, dissimilar context C remains low. The increase in fear generalization occurs because, as soon as the recall of context B is driven by the long-term CTX encoding (overlapping with the CTX encoding of context A) rather than the transient HIP engram, the BA_N module converges to its representation of context A.

We varied the following parameters, affecting the model's fear expression, long-term maintenance of fear associations (via synaptic homeostasis), as well as recurrent excitation in BA_N:

- θ_P (BA_P firing threshold): uniform in $[2.8, 3.3]$,
- T^P_{ext} : The *extinction threshold* of the homeostasis rule – the synaptic strength below which $BA_N \to BA_P$ synapses are pruned during *Sleep*,
- recurrent excitatory gain g_L of BA_N during *Recall*: log-uniform in $[0.01, 0.75]$ – affecting the importance of excitation within BA_N relative to plastic inputs from HIP or CTX.
- τ_L^{slow} of BA_N: Learning rate of recurrent, excitatory synapses in BA_N in the absence of emotionally significant events – affecting the longevity of BA_N engrams.
- A_P : The *recruitment level* of the homeostasis rule acting on $BA_N \to BA_P$ synapses during *Sleep*, i.e., the target strength of $BA_N \to BA_P$ synapses consolidated by this process.

We performed Latin Hypercube Sampling (LHS) with 50 parameter sets and 10 independent noise seeds per set. We chose a smaller number of samples relative to the previous stability analysis due to the length of the simulation protocol – as described for Fig 4d. Averaging across the 10 noise seeds yielded three time series A_t, B_t, C_t for $t = 0, \cdots, 15$, denoting fear expression during the *Recall* of each context after t *Perception-Sleep* cycles.

Any parameter setting was marked as a PASS if it satisfied the three following **pass/fail criteria**:

- **Fear in the conditioned context remains high by the end of the simulation:** $A_{15} \geq 0.5$,
- **No fear is expressed in C at any time:** $\max_t C_t \leq 0.3$,
- **Generalized fear in B increases over time:** $B_0 < A_0, B_{15} \geq 0.5$ and $\text{Spearman}(t, B_t) \geq 0.25$.

Fig D summarizes the prevalence of these three outcomes across the 50 parameter settings. The 'C_low' criterion was the most robust – fear expression in C occurred just once. The 'A_high' criterion was satisfied in 19 of the 50 settings – as shown in Fig E, lower values of θ_P and T^P_{ext} were generally helpful for ensuring that the model held on to the fear association acquired in context A over the course of the simulations. Third, the 'B_increase' criterion was the trickiest to satisfy; it held only for 6 of the 50 parameter samples. For many parameter combinations, fear either generalized from A to B immediately after conditioning or, more often, failed to generalize by the end of the simulation. As per Fig F, this criterion could only be met with low values of θ_P and

T^P_{ext}. These results indicate that our model's ability to remember fear memories over the course of many *Perception-Sleep* cycles relies on a relatively delicate parameter balance – compared to earlier results on fear generalization independent from *Sleep*. More concretely, if the *extinction threshold* of the homeostasis rule is set too high, $BA_N \rightarrow BA_P$ synapses that encode a fear association may be prematurely pruned, causing fear to be unlearned.

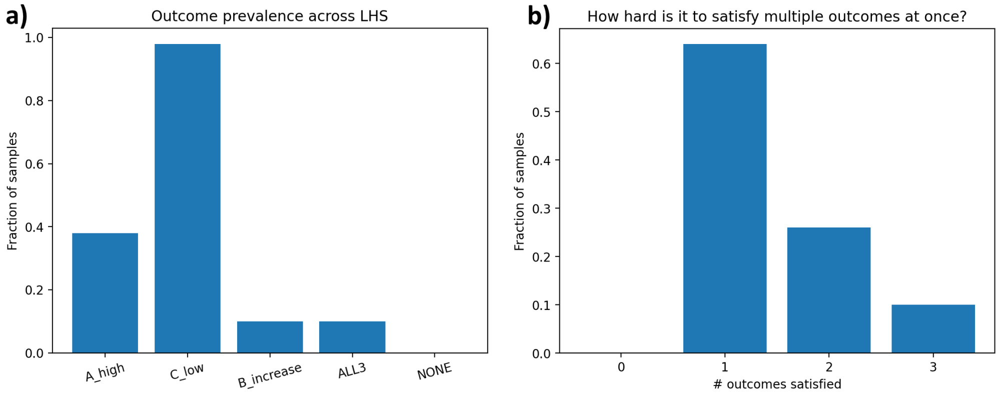

Fig D. Stability Analysis – generalization increases over time: outcome prevalence and combinations.
a): fraction of LHS samples that satisfy each individual outcome (A_high, C_low, B_increase) and all three simultaneously (ALL3).
b): distribution over the number of outcomes satisfied per sample (0–3). Results shown for $n = 50$ LHS settings with $m = 10$ seeds per setting.

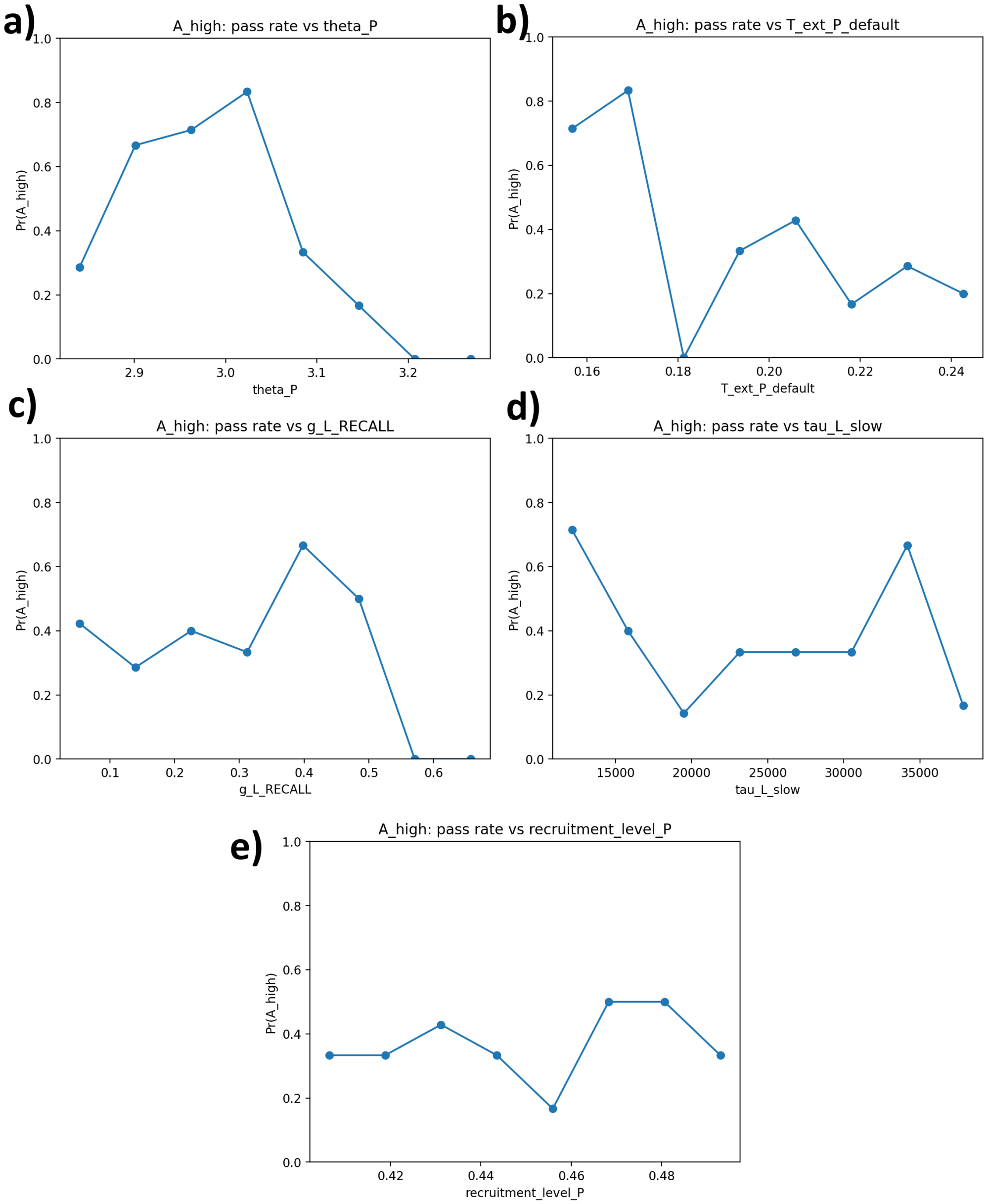

Fig E. Stability Analysis – generalization increases over time: binned pass rates for 'A high' criterion. For each parameter, the LHS samples ($n = 50$) are split into 8 equal-width bins; the y-axis shows the fraction of settings in each bin that satisfy the criterion that 'fear in A remains high by the end of the simulation'.

Panels:
a) θ_P;
b) T_{ext}^{P};
c) g_L of BA_N during *Recall*;
d) τ_L^{slow} of BA_N;
e) A_P.

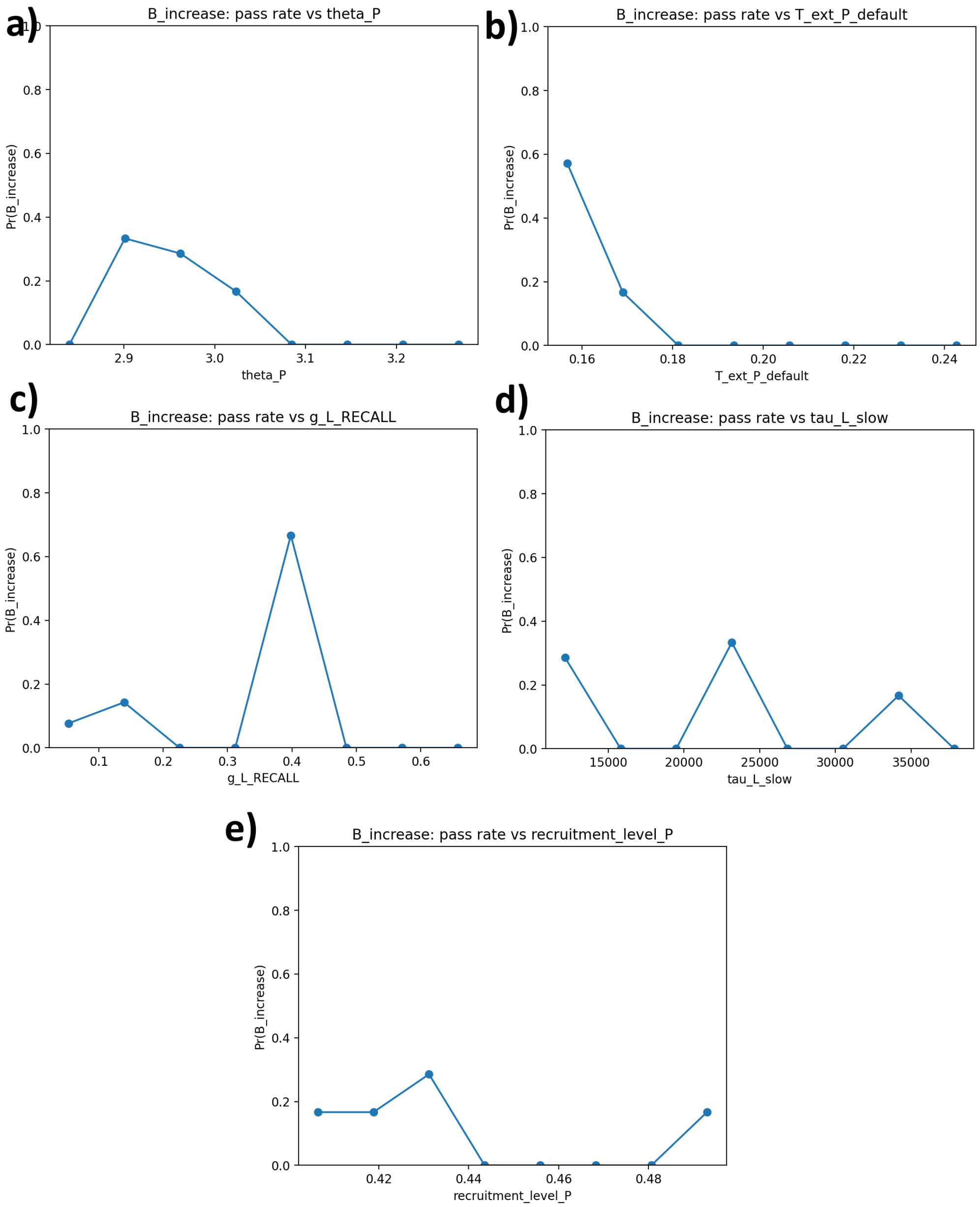

Fig F. Stability Analysis – generalization increases over time: binned pass rates for 'B increase' criterion. As in Fig E, but the pass condition is that 'generalized fear in B increases over time'.

Disrupting sleep causes fear sensitization

In our main results, we demonstrate that shortening (or omitting) our model's *Sleep* phase over an extended time period, during which contexts and US signals of varying strength are presented, causes *fear sensitization*. Concretely, Fig 8 shows that the model becomes more prone to acquiring fear *rapidly* in a novel, aversive context – an effect that is explained by accumulated increases in $BA_N \to BA_P$ synapse strength due to a lack of synaptic homeostasis.

To evaluate the robustness of this result, we varied the following parameters in our stability analysis:

- θ_P (BA_P firing threshold): uniform in $[2.8, 3.3]$,
- recurrent excitatory gain g_L of BA_N during *Recall*: log-uniform in $[0.01, 0.75]$,
- T^P_{ext} : The *extinction threshold* of the homeostasis rule acting on $BA_N \to BA_P$ synapses,
- τ_L^{slow} of BA_N: Learning rate of recurrent, excitatory synapses in BA_N in the absence of emotionally significant events,
- τ_L^{fast} of BA_N: Learning rate of recurrent, excitatory synapses in BA_N *during* emotionally significant events,
- τ_{FB}^{fast} of $BA_N \to BA_P$: Learning rate of fear-(association-)coding synapses during surprising US signals.

We performed Latin Hypercube Sampling (LHS) with 50 parameter sets and 10 independent noise seeds per set. The simulation protocol we used was as described for Fig 8 – with the exception that we limited the selection of values for the 'fraction of omitted sleep' to $f = 0.0, 0.33, 0.67, 1.0$. Averaging across the 10 noise seeds thus yielded four values of $C(f)$ – the amount of fear expressed after 5 time steps in the novel context at the end of the simulation – and four values of $W(f)$ – the summed synaptic weight of $BA_N \to BA_P$ synapses – for each LHS sample. Within each sample, the four different model instances were subjected to the same weight initialization at the start of the simulation, and observed the same contexts and US signals over its course.

For each parameter set, we assessed two simple **pass/fail criteria**:

- **The $BA_N \to BA_P$ synaptic density increases with sleep omission:** $W(0) \leq W(0.33) \leq W(0.67) \leq W(1)$,
- **Fear acquisition in the novel context increases with sleep omission:** $C(0) \leq C(0.33) \leq C(0.67) \leq C(1)$,

As per Fig G, the first criterion held for all 50 samples, showing that – irrespective of precise parameter values – *Sleep* predominantly induces a synaptic *weakening* in our model. Although it is intuitively expected that a larger net $BA_N \to BA_P$ will *generally* be linked with increased fear expression/acquisition in our model, the second criterion only held for 16 of the 50 samples. We expect that the main reason underlying this discrepancy is the increased stochasticity of fear acquistion in our model, since different model instances within each LHS sample will have different sets of 'highly recruitable' BA_P cells towards the end of the simulation – so that, when averaging across just 5 noise seeds, curve C was not always monotonously increasing. To gain confidence that sleep disruption yet *generally* increases fear sensitization across different parameter sets, Fig H shows the average W and C curves across all 50 samples; both are strictly increasing.

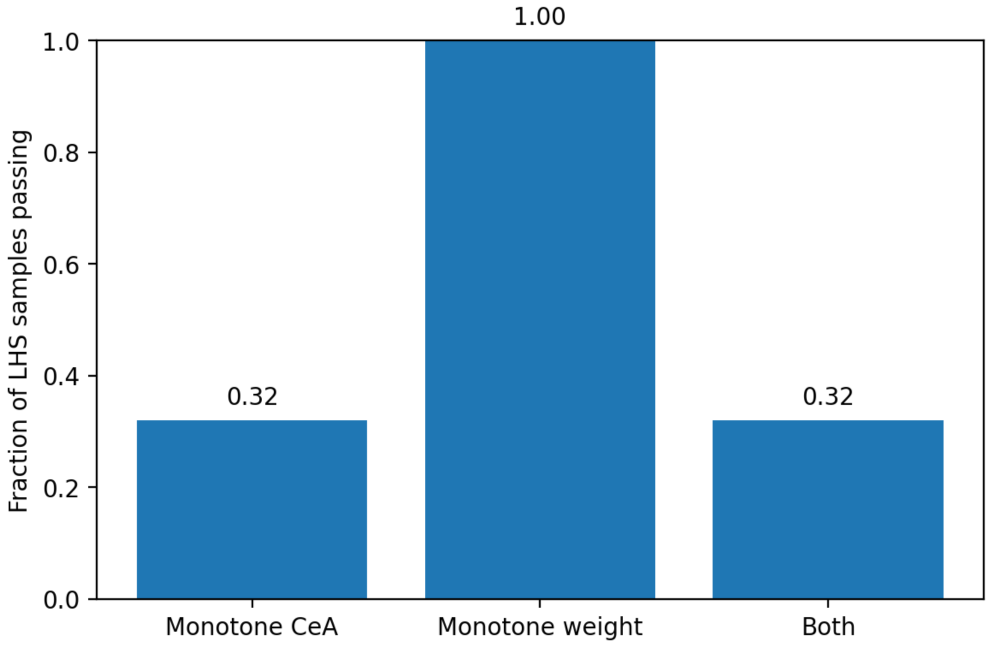

Fig G. Stability Analysis – sleep disruption: pass rates. Acceptance rates across 50 LHS samples, for the two pass/fail criteria described in the stability analysis on sleep disruptions. The summed $BA_N \to BA_P$ synaptic weight consistently increased with sleep omission, whereas the monotonicity criterion on fear acquisition in the novel context was only satisfied in 16 out of 50 cases.

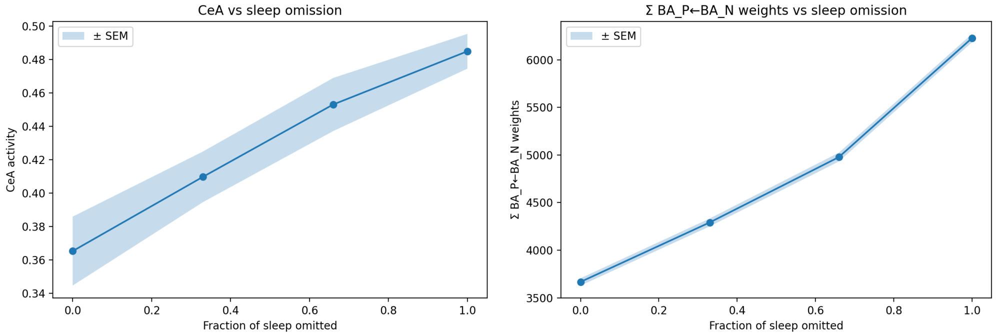

Fig H. Stability Analysis – sleep disruption: average outcomes across LHS samples. We averaged the output curves of our sleep disruption experiment – fear acquired in the novel context and total $BA_N \to BA_P$ synaptic weight for different levels of sleep omission – across all 50 LHS samples. Although, as per Fig G, fear acquisition was only strictly increasing with sleep omission for 16 of 50 parameter sets, the criterion held for the 'grand average'. Shaded bands denote one standard error on the mean.

Long-term effects of stress-induced fear sensitization

In the final section of our main results, we demonstrate the effects of temporary increases in BA_P recruitability and the $BA_N \to BA_P$ extinction threshold, T^P_{ext}. Fig 9c and 9d demonstrate that, although these changes – which are made to occur when the model is exposed to an extremely strong US signal for a sufficient amount of time – are transient, they have a long-lasting impact on the model's total $BA_N \to BA_P$ synapse strength and tendency towards acquiring fear in novel contexts. That is because synapses $BA_N \to BA_P$ formed and consolidated while the stress-induced changes are active are later preserved by the homeostasis rule, even after T^P_{ext} has returned to its default.

To show that this effect does not depend on a specific parameter configuration, we performed a stability analysis, randomly sampling the same parameters as in the previous analysis on sleep disruptions (with the same parameter ranges), in addition to the following:

- $T^P_{\text{ext, min}}$: The value to which the *extinction threshold* of the $BA_N \to BA_P$ homeostasis rule is lowered after the model encounters an extreme US signal: uniform in $[0.075, 0.125]$.
- $T^{P,\text{recovery}}_{\text{ext}}$: The rate per time step at which the *extinction threshold* recovers to its default value T^P_{ext} after being reduced: uniform in $[\frac{1}{40{,}000}, \frac{1}{10{,}000}]$.

We performed Latin Hypercube Sampling (LHS) with 50 parameter sets and 5 independent noise seeds per set. The simulation protocol we used was as described for Fig 9c. In short, on each run of the simulation, two model instances were initialized. One of them was exposed to an extremely strong ('traumatic') US signal, activating stress-induced parameter changes as described. Afterwards, both model instances were exposed to 15 *Perception-Sleep* cycles, encountering 4 random, moderately aversive contexts each day. At the end of the simulation, both model instances were briefly exposed to a strong US signal in a novel context to test the amount of fear they acquired after 4 time steps. Similar to the previous analysis, this protocol yielded fear expression values C_{control} and C_{trauma} and summed $BA_N \to BA_P$ synaptic weights W_{control} and W_{trauma}. The values of these output measures were averaged across the 5 noise seeds.

We assessed two simple **pass/fail criteria** for each set of sampled parameters:

- The 'traumatized' model had a greater summed strength of $BA_N \to BA_P$ synapses, $W_{\text{trauma}} > W_{\text{control}}$.
- The 'traumatized' model acquired more fear in the novel context, $C_{\text{trauma}} > C_{\text{control}}$.

Similarly to the previous analysis on sleep disruptions, the first criterion held for all 50 test cases, as per Fig I. This is not surprising, since the stress-induced parameter changes strongly favour both the formation and preservation of $BA_N \to BA_P$ synapses for about 5 simulated days following exposure to the traumatic stressor – an increase in net synapse strength that is preserved by the model's homeostasis rule once those synapses have consolidated. The second criterion reached a pass rate of 44%, showing that a higher synaptic density in the $BA_N \to BA_P$ *generally* – though not *always* – leads to accelerated fear acquisition in novel contexts.

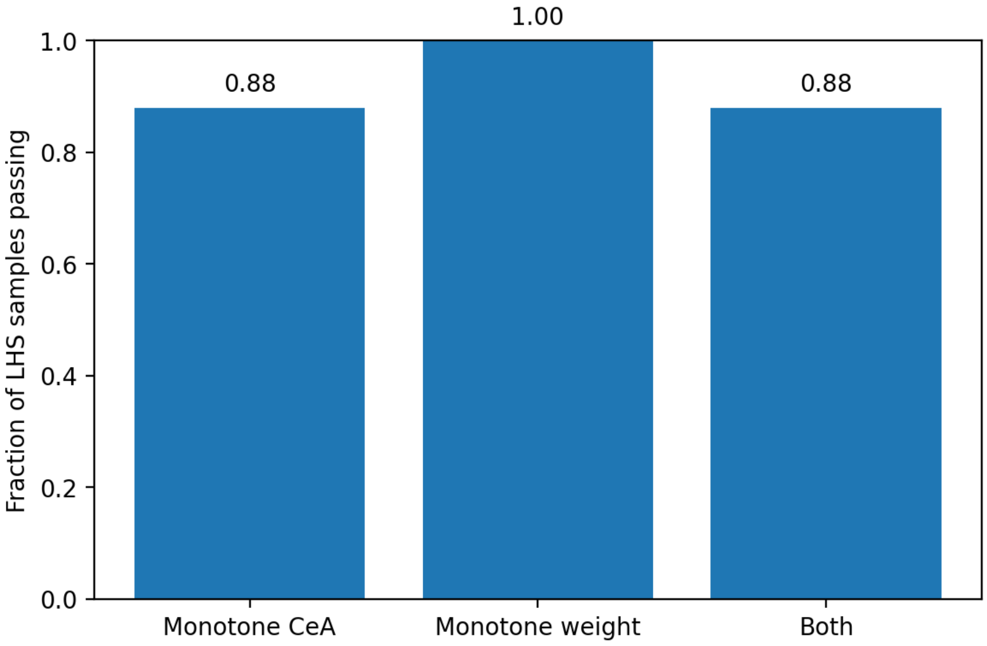

Fig I. Stability Analysis – SEFL: pass rates. Acceptance rates across 50 LHS samples, for the two pass/fail criteria described in the stability analysis on stress-enhanced fear learning. For all 50 parameter samples, the summed $BA_N \rightarrow BA_P$ synaptic weight, averaged across 5 noise seeds, was consistently larger for a model exposed to a 'traumatic' US 15 days prior. In 44 of the samples, the 'traumatized' model consequentially acquired more fear upon brief exposure to a novel context paired with a strong US signal, relative to a control model with the same prior history, except for the 'traumatic' event.

References

1. Loh WL. On Latin hypercube sampling. The annals of statistics. 1996;24(5):2058-80.
