# Supplementary material for: Learning, sleep replay and consolidation of contextual fear memories: A neural network model
Source: PLoS Comput Biol. 2026 Mar 17;22(3):e1013251. doi: 10.1371/journal.pcbi.1013251 (PMC13012624; doi:10.1371/journal.pcbi.1013251)
Supplement: S4 Appendix — (PDF) [file pcbi.1013251.s004.pdf]

S4 Appendix Simulation Protocols

Model phase	No. time steps	Context Input	US Input	Comment
Perception	20	A	0.0	Pre-exposure.
Perception	30	A	1.0	Acquisition.

Table A. Demonstrating engram formation. Fig 4a.

Model phase	No. time steps	Context Input	US Input	Comment
Perception	30 per context	1 to 5	0.0	–
Perception	30 per context	6 to 10	0.5	Mild US to engage BA.
Sleep	165	–	0.0	–

Table B. Demonstrating sleep replay. Fig 4b.

Model phase	No. time steps	Context Input	US Input	Comment
Repeated for 21 simulated days				
Perception	120 (split between con-texts)	Four new contexts per day.	0.5 for first 4 steps, then 0.0.	–
Sleep	165	–	0.0	–
After final day:				
Recall	31 per context (1 + 30).	All stored contexts (10% noise).	0.0	Context shown for 1 step, then 30 steps without input (net-work converges).

Table C. Assessing recall performance. Fig 4c. To simulate a scenario where some contexts are more likely to be remembered than others, the presentation lengths of the four contexts shown on any day are partitioned from a total of 120 time steps using a random allocation procedure. To engage the BA_N module, each input is paired with a moderate US of strength 0.5 for the first 4 steps of its presentation. During *Recall* of a context, the original input pattern (with 10% of its bits flipped) is presented for a single time step, followed by 30 steps without further input, allowing the model to converge. For each of HIP, CTX and BA_N, a recall distance d is computed by comparing the module's current output vector $\mathbf{u}$ to the activity $\mathbf{v}$ it had when the context was first presented during *Perception*. Recall is successful if $d(u, v) = \frac{1-\mathbf{u}\cdot\mathbf{v}}{2|\mathbf{u}||\mathbf{v}|} < 0.15$ [1, 2].

**Model phase**	**No. time steps**	**Context Input**	**US Input**	**Comment**
Perception	20	A	0.9	Acquisition.
Perception	50	A	0.0	Extinction.

Table D. Basic 'AA' fear extinction. Fig 4d.

**Model phase**	**No. time steps**	**Context Input**	**US Input**	**Comment**
Repeated for 20 acquisition steps				
Perception	1	A	0.9	Acquisition step.
Recall	1 per context	Similarity contexts B (with Overlap $A \leftrightarrow B \in \{0.0, 0.2, 0.4, 0.5, 0.6, 0.7, 0.75, 0.8, 0.85, 0.9, 0.925, 0.95, 0.975\}$), as well as A.	0.0	After each step of fear acquisition in context A, probe BA_P, BA_I, and CeA responses when exposed to contexts of varying similarity to A.
Repeated for 50 extinction steps				
Perception	1	A	0.0	Extinction step.
Recall	1 per context	Similarity contexts B (same set as above) and A.	0.0	Same test procedure as during acquisition.

Table E. Generalization gradients of fear and extinction. After each Perception step in A, the model is switched to *Recall* so that fear expression can be probed for one step in each similarity context (13 values listed), as well as in A. Fig 5b.

Model phase	No. time steps	Context Input	US Input	Comment
**ABA renewal**				
Perception	20	A	1.0	Acquisition.
Perception	100	B	0.0	Extinction.
Perception	100	A	0.0	Renewal and re-extinction.
**ABC renewal**				
Perception	20	A	1.0	Acquisition.
Perception	100	B	0.0	Extinction.
Perception	100	C	0.0	Renewal and re-extinction.
**AAB renewal**				
Perception	20	A	1.0	Acquisition.
Perception	100	A	0.0	Extinction.
Perception	100	B	0.0	Renewal and re-extinction.

Table F. 'ABA', 'ABC' and 'AAB' fear renewal protocols. In all cases, B (and C for ABC) are generated to be similar to A with an $A \leftrightarrow B$ (and $A \leftrightarrow C$) overlap of 80% in the input pattern. Fig 5c.

Model phase	No. time steps	Context Input	US Input	Comment
**Exposure to contexts A, B and C, with fear acquisition in A**				
Perception	25	A	0.9	Conditioning.
Perception	25	B ($A \leftrightarrow B = 0.6$)	0.0	–
Perception	25	C ($A \leftrightarrow C = 0.0$)	0.0	–
Recall	$1 + 30$	A, B, C	0.0	Each context shown for 1 step, followed by 30 steps without input.
Sleep	165	–	0.0	–
**Delay period (15 days, repeated)**				
Perception	3×25	3 random, novel contexts	$0.9 \cdot \text{Beta}(1.25, 5)$ per context	Background exposure.
Recall	$1 + 30$	A, B, C	0.0	Each context shown for 1 step, followed by 30 steps without input.
Sleep	165	–	0.0	–

Table G. Generalisation after consolidation. Fig 5d.

**Model phase**	**No. time steps**	**Context Input**	**US Input**	**Comment**
**Fear acquisition**				
Perception	20	A	0.9	Acquisition in A.
**Single-context extinction arm**				
Perception	100	$B(A \leftrightarrow B = 0.8)$	0.0	Extinction in single B.
Perception	100	$C(A \leftrightarrow C = 0.8)$	0.0	Renewal and re-extinction.
**Multi-context extinction arm**				
Perception	$100(4 \times 25)$	$B_1, \ldots, B_4(A \leftrightarrow B_i = 0.8)$	0.0	Extinction distributed across 4 contexts (even split).
Perception	100	$C(A \leftrightarrow C = 0.8)$	0.0	Renewal and re-extinction.

Table H. Single- vs multi-context extinction. After the acquisition phase, the model is cloned and separately exposed to the single- and multi-context protocols to allow for a fair comparison (Fig 6a). *Fig 6b* uses the same protocol but *only the multi-context arm.* The plot reports CeA at the *first* renewal step in C as a function of $A \leftrightarrow B$ (11 values from 0.50 to 1.00), repeated across $A \leftrightarrow C \in \{0.60, 0.70, 0.80, 0.90\}$.

**Model phase**	**No. time steps**	**Context Input**	**US Input**	**Comment**
Perception	40	A	0.9	Acquisition.
Perception	40	B (A↔B = 0.5)	0.0	No conditioning.
Sleep	165	–	0.0	Homeostasis.
Perception	2	A	0.0	Post-sleep probe (2 steps).
Perception	2	B	0.0	Post-sleep probe (2 steps).

Table I. Effect of homeostasis on P-cell recruitment strength. Fig 7.

Model phase	No. time steps	Context Input	US Input	Comment
**Repeat for 7 simulated days**				
Perception	3×20 per day	3 random, novel contexts each day	$0.85 \cdot \mathrm{Beta}(1, 1)$ per context	Daily experiences.
Sleep	$165 \times (1 - f)$	–	0.0	Sleep length scaled by omission fraction $f \in \{0, 0.1, \cdots, 1.0\}$.
**Probe acquisition in A**				
Perception	3	A	0.75	Light acquisition.

Table J. Sleep deprivation. For each model (sleep omission fraction f), 7 days of background exposures are followed by light acquisition in A; the resulting fear output and net $BA_N \rightarrow BA_P$ synaptic weight is recorded. Fig 8.

Model phase	No. time steps	Context Input	US Input	Comment
Perception	30	A	1.0 (0.0 in controls)	Traumatic stress.
Perception	2×30	Random contexts	$0.9{\cdot}\mathrm{Beta}(1, 2.5)$	Fairly harmless.
Sleep	165	–	0.0	–
Perception	3	B	0.6	Moderate US.
Perception	27	B	0.0	–
Perception	2×30	Random contexts	$0.9{\cdot}\mathrm{Beta}(1, 2.5)$	Fairly harmless.
Sleep	165	–	0.0	–
Recall	30	B (on 1st step)	0.0	–

Table K. SEFL protocol. Exposure to harmless contexts 'C' and 'D' is included to 'balance out' hippocampal replay during the following *Sleep* phase. Fig 9a.

Model phase	No. time steps	Context Input	US Input	Comment
Perception	3	B	0.6	Moderate US.
Perception	27	B	0.0	–
Perception	2×30	Random contexts	$0.9{\cdot}\mathrm{Beta}(1, 2.5)$	Fairly harmless.
Sleep	165	–	0.0	–
Perception	30	A	1.0 (0.0 in controls)	Traumatic stress.
Perception	3×30	Random contexts	$0.9{\cdot}\mathrm{Beta}(1, 2.5)$	Fairly harmless.
Sleep	165	–	0.0	–
Recall	30	B (on 1st step)	0.0	–

Table L. SEFL protocol — Order reversed. Same as Table K, but with the trauma and moderate-US components swapped. Fig 9b.

**Model phase**	**No. time steps**	**Context Input**	**US Input**	**Comment**
**Traumatic stressor (extreme US delivery)**				
Perception	30	A	1.0 (0.0 in controls)	Traumatic stress.
Perception	2×30	Random contexts	$0.9 \cdot \mathrm{Beta}(1, 2.5)$	Fairly harmless.
Sleep	165	–	0.0	–
**15 delay days (3 random contexts/day)**				
Perception	3×30 per day	Random	$0.9 \cdot \mathrm{Beta}(1, 2.5)$ per context	Moderate fear exposure over time.
Sleep	165	–	0.0	–
**Assessing novel fear acquisition**				
Perception	3	B	0.85	Moderate US.
Perception	27	B	0.0	–
Perception	2×30	Random contexts	$0.9 \cdot \mathrm{Beta}(1, 2.5)$	Fairly harmless.
Sleep	165	–	0.0	–
Recall	30	B (on 1st step)	0.0	–

Table M. SEFL protocol – Delayed Sensitization test. Same as Table K, but with 15 'delay days', on each of which three random contexts (of moderate fearfulness) are presented. After those 15 days, 'moderate fear acquisition' in context 'B' is performed as in the default SEFL protocol. Fig 9c and 9d.

References

1. Greve A, Donaldson DI, Van Rossum MC. A single-trace dual-process model of episodic memory: A novel computational account of familiarity and recollection. Hippocampus. 2010;20(2):235-51.

2. Fiebig F, Lansner A. Memory consolidation from seconds to weeks: a three-stage neural network model with autonomous reinstatement dynamics. Frontiers in computational neuroscience. 2014;8:64.
