# Supplementary material for: Learning, sleep replay and consolidation of contextual fear memories: A neural network model
Source: PLoS Comput Biol. 2026 Mar 17;22(3):e1013251. doi: 10.1371/journal.pcbi.1013251 (PMC13012624; doi:10.1371/journal.pcbi.1013251)
Supplement: S1 Text — (PDF) [file pcbi.1013251.s008.pdf]

Our model assumes that fear extinction is more context-specific than contextual fear itself. If this is the case, extinguishing fear in a conditioned context (A) does not abolish fear generalization. Extinction in multiple contexts – not too similar and not too dissimilar to A – may be a more effective strategy.

To illustrate the intuition behind this prediction, here we define a toy model. Suppose contextual inputs correspond to points in the plane. Define context A as the origin; represent any other context B with similarity s to A as a point with distance $1 - s$ from the origin. Assume that fear acquisition produces a broad two–dimensional Gaussian "fear" field centred at A ($\sigma = 0.45$). Further assume that extinction in any context B produces a narrower Gaussian field, centered at the corresponding location ($\sigma = 0.18$). Then, define net fear expression in context C as $\mathrm{ReLU}(\mathrm{Acq(C)} - \mathrm{Ext(C)})$, where Acq denotes the acquisition field and Ext denotes the sum of all extinction fields.

This toy model is independent from our actual implementation, but similarly captures the assumption that extinction generalizes to a narrower range of environments than fear itself.

In the following experiments, we define extinction contexts B_1 to B_4 with angles of $0, \pi/2, \pi$ and $3\pi/2$, relative to the origin. Renewal context C is chosen with an angle of $5\pi/4$ and a fairly large similarity to A ($s = 0.8$).

In panel a of Fig A below, the extinction contexts are very similar to A (with $s = 0.95$). Thus, the extinction fields are tightly clustered around A. Fear expression at A is extinguished, but there is a range of contexts, including C, to which fear generalizes but extinction does not.

In panel b, the extinction contexts are fairly similar to A ($s = 0.85$). The extinction fields do not entirely abolish fear expression in A itself but, collectively, they now cover most of the acquisition field. As a result, fear expression in context C is lowered significantly.

In panel c, the extinction contexts are quite dissimilar from A ($s = 0.65$). Importantly, the extinction fields themselves do not overlap. Therefore, extinction in each B_i only affects fear expression in a narrow range of similar contexts, but there is no cumulative effect. Fear expression remains fully intact in a large portion of the acquisition field, including C.

Panel d illustrates fear expression in C as a function of the $A - B_i$ similarity. As indicated above, an intermediate similarity around $0.8 - 0.9$ minimizes fear renewal in C. Interestingly, the shape of this schematic qualitatively matches the shape of the curves in Fig 6b of our main results.

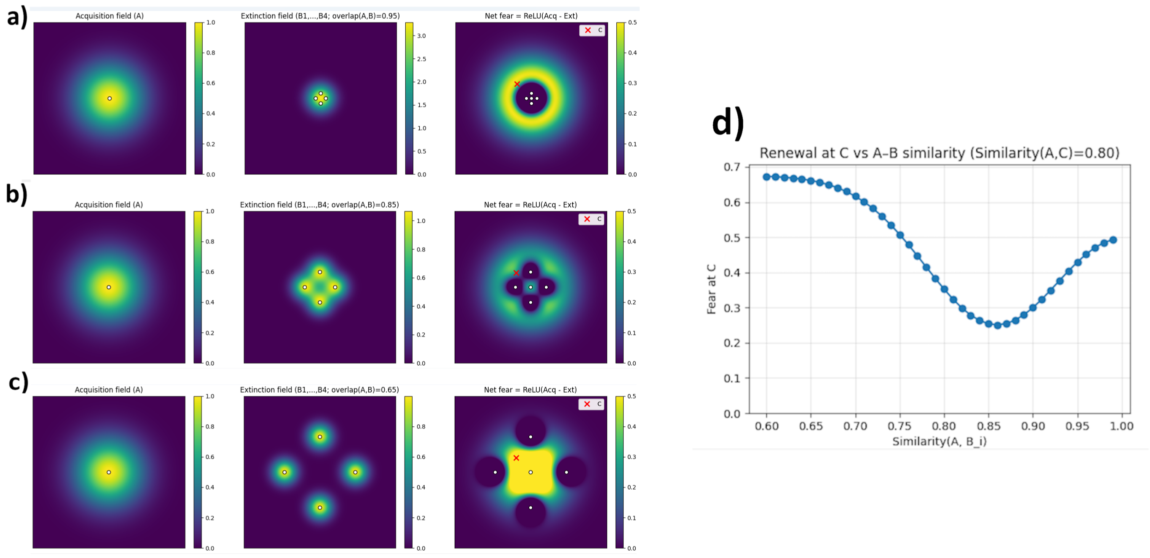

Fig A. An intermediate similarity between acquisition and extinction contexts minimizes fear renewal in a toy model.

Panels a–c show, from left to right, the acquisition field, extinction fields, and resulting fear expression, for the toy experiments described above. In each panel, extinction contexts B_i are placed at angles 0, $\pi/2$, π, and $3\pi/2$ relative to A, with varying similarity between A and the B_i: 0.95 in a), 0.85 in b), and 0.65 in c). The location of the renewal context C (with an A-similarity of 0.8) is marked with a red cross in the rightmost panels.

Panel d shows fear expression in C as a function of the similarity s between the acquisition and extinction contexts. Fear renewal is minimized for $s \approx 0.8$–0.9.
