# Supplementary material for: Learning, sleep replay and consolidation of contextual fear memories: A neural network model
Source: PLoS Comput Biol. 2026 Mar 17;22(3):e1013251. doi: 10.1371/journal.pcbi.1013251 (PMC13012624; doi:10.1371/journal.pcbi.1013251)
Supplement: S2 Text — (PDF) [file pcbi.1013251.s009.pdf]

In our main results section on sleep deprivation, we subjected our model to 7 days filled with exposure to various contexts, joined by random, generally moderate US signals. At the end of the simulation, we evaluated the amount of fear expressed by the model after the delivery of three US signals in a novel environment. Based on this protocol, Fig 8 demonstrates that shortening, or completely omitting, the model's daily *Sleep* phase leads to increases in fear acquisition.

In this supplementary simulation, we repeated this protocol for instances of the model subjected to the following alterations of the homeostasis rule acting on $BA_N \to BA_P$ synapses during *Sleep*:

- Multiplying or dividing the rate of homeostatic synaptic change by 5.
- Lowering the *extinction threshold* T^P_{ext} of the $BA_N \to BA_P$ homeostasis rule, from 0.175 to 0.10. This means that $BA_N \to BA_P$ have to be less strong to be preserved overnight. In other words, overnight synaptic pruning becomes less aggressive.

Simulation outcomes are shown below, in Fig A below.

For the first two variants, fear acquisition at the end of the simulation continues to be inversely proportional to the duration of *Sleep*. A faster or slower homeostasis rate, respectively, however decreases, or increases, fear acquisition. Thus, accelerated homeostasis is able to compensate for shortened *Sleep* in our simulations, at least in this regard.

For the third variant, an extended *Sleep* length interestingly no longer lowers fear acquisition, but rather results in a slight increase relative to shorter durations. That is because the altered homeostasis rule makes moderately weak $BA_N \to BA_P$ synapses more likely to be gradually strengthened, rather than pruned, throughout *Sleep*. However, completely omitting *Sleep*, thereby preventing an overnight modulation of $BA_N \to BA_P$ synapses altogether, still causes the largest amount of fear sensitization.

In conclusion, these outcomes underline the finding that the moderating effect of *Sleep* on later fear acquisition relies on a homeostasis weakening of fear-coding synapses.

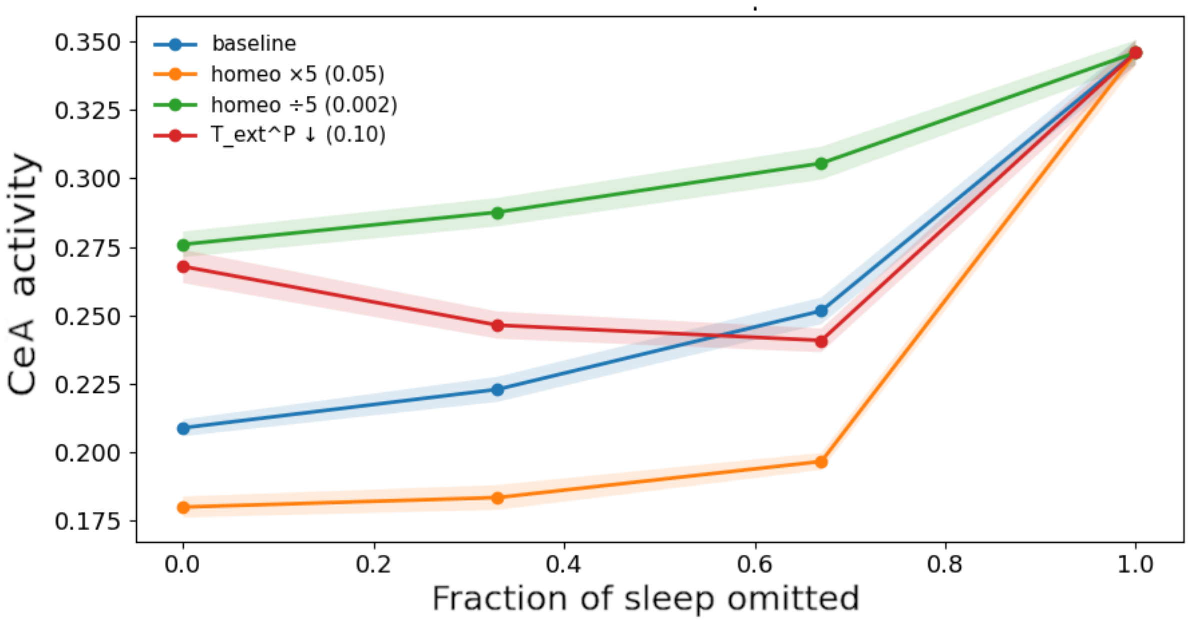

Fig A. Effects of sleep deprivation for different model variants.
As discussed above, different variants of our model were subjected to 7 days filled with exposure to various contexts, before being briefly tested for fear acquisition in a novel environment. The model's CeA output was recorded after 3 time steps of US delivery (strength 0.6).
Dots denote the mean, shaded regions one standard error across 200 runs of the simulation.
For the default model configuration ('baseline'), as well as for the variants with accelerated ('homeo ×5') or decelerated ('homeo ÷5') homeostatic changes, fear acquisition scaled inversely with *Sleep* duration. For the variant with a lowered *extinction threshold* ('T_ext^P'), longer *Sleep* durations no longer had this moderating effect.
Note that, when 100% of *Sleep* were omitted, the different model instances behaved identically, because we used random seeds to prevent any stochastic differences in terms of context generation, weight initialization or cell recruitability.
