# Supplementary material for: Learning, sleep replay and consolidation of contextual fear memories: A neural network model
Source: PLoS Comput Biol. 2026 Mar 17;22(3):e1013251. doi: 10.1371/journal.pcbi.1013251 (PMC13012624; doi:10.1371/journal.pcbi.1013251)
Supplement: Attachment — Submitted filename: Reply_to_PLOS_Reviewers-final.pdf [file pcbi.1013251.s010.pdf]

Learning, sleep replay and consolidation of contextual fear memories: A neural network model – Reply to reviewers

Lars Werne, Angus Chadwick, Peggy Seriès

November 2025

1 Summary

We sincerely thank the reviewers for their time and efforts. We have made several changes to our manuscript, in line with the reviewers' recommendations, including the following:

- We now discuss additional empirical results from recent fear conditioning experiments, suggest interpretations of those findings through the lens of our model or, where relevant, suggest extensions of our model that may be suitable for studying the underlying mechanisms in greater detail.
- We have added a novel simulation result, which highlights a consequence of our model's extinction mechanism in greater detail, and may inspire experimental procedures for clarifying the circumstances under which *fear extinction in multiple contexts* can be beneficial.
- We include a stability analysis assessing the robustness of the main qualitative simulation outcomes reported in our results.
- We have nuanced our description of the model's results, in order to avoid over-interpretation, being more transparent about its limitations and intended level of abstraction.

In the following, we respond to the reviewers' comments in greater detail. Comments are in *italics*; our responses are in normal typeface. Labels such as R1-Maj1 and R2-Min3 indicate reviewer and comment number.

2 Reviewer 1

The paper presents a biologically grounded neural network model of contextual fear memory formation and consolidation, organized into modules that mirror key brain regions. A sensory cortical module encodes environmental contexts and

sends this information in parallel to both a hippocampal module, which encodes contexts rapidly via entorhinal inputs, and a neocortical module that gradually builds long term, generalized context representations through consolidation. A basal amygdala module encodes associations reflecting each context's valence. Offline replay allows these representations to be strengthened in the neocortex. Replay supports both renewal and time dependent increases in generalization, while synaptic homeostasis trims weak associations to prevent overgeneralization. These dynamics also enable the model to account for stress enhanced fear learning and the maladaptive effects of sleep deprivation.

The manuscript is clear and well-organized, outlining each model component and its empirical rationale in a way that allows readers to understand how replay and synaptic downscaling contribute to the observed effects. The model offers a thorough, operationalized implementation of the hypothesis that fear generalization reflects hippocampal-to-neocortical transfer, providing a strong foundation for future experimental work.

We thank the reviewer for the positive comments.

However, the conceptual novelty of the framework may be somewhat limited, as systems consolidation has long been proposed as a mechanism underlying generalization.

We agree that the putative link between systems consolidation and decreased memory specificity is not conceptually novel, as many authors have argued in its favour (e.g. [1, 2, 3]). Implementing this concept in a neural architecture however did pose some novel questions, for instance: If hippocampal and cortical memory representations of the same event can both attain a valence by recruiting *the same* ensemble of downstream amygdala cells [4], then how is this association transferred? The offline co-activation of hippocampal, cortical and amygdalar engrams proposed by our model may be an 'obvious' candidate mechanism in light of these *existing* conceptions. We argue that, nevertheless, proposing a model that implements this hypothesis is useful; not least because it can serve as a starting point for experiments investigating details of the way, or alternative ways, in which the brain may implement this process.

The following comments raise several issues regarding the rationale behind specific architectural choices, the consistency of mechanistic assumptions across simulations, and the model's alignment with related empirical findings on contextual fear memory consolidation.

2.1 Major comments: [R1-Maj]

R1-Maj1

There appear to be discrepancies between the model's architecture and established anatomical pathways in the brain. In the model, the sensory cortex feeds separately into the hippocampus and neocortex. However, in the brain, the sensory cortex is part of the neocortex, and hippocampus inputs are processed through neocortical hierarchies that interact with the hippocampus via a bidirectional loop. The inclusion of ECout as an isolated output in the model also departs from the brain, as the entorhinal cortex receives hippocampal outputs and projects them back to the neocortex. The authors should clarify or revise these architectural choices to better reflect the relevant circuitry.

We acknowledge that some confusion could be caused by the naming of our model's separate 'CTX' and 'SC' modules, given the fact that, anatomically, the sensory cortex belongs to the neocortex. We would like to clarify that – despite the naming, which was borrowed from Fiebig & Lansner (2014; [5]) – the 'CTX' module is not intended to represent the entire neocortex, but a hypothetical population of context-coding engram cells in the neocortex, as has been identified in the prefrontal cortex for contextual fear memories [4]. Similarly, our 'ECin' and 'ECout' modules serve the computational task of – during *Recall* – determining whether HIP was able to retrieve a memory matching the current environmental input (cf. Krasne et al., 2015; [6]) and modulating the synaptic gain of afferents to the amygdala accordingly. Here, we did not aim to account for EC's full set of responsibilities. To defuse this potential source of confusion, we have added the following to our model description:

'Its modules represent hypothetical populations of neurons with narrowly defined computational roles. They are named after the brain regions assumed to accommodate neurons with those roles, but the model is not intended to capture the full cellular heterogeneity or connectivity of the implicated regions.'

Our model does not serve as a complete connectome, since it deliberately abstracts away from known, or highly plausible, anatomical pathways for the sake of conceptual clarity. For instance, the model's synapses from *HIP* to *CTX* correspond to a putative functional connection that could allow re-activation of a hippocampal engram to drive the concurrent replay in the neocortex. Our model is not intended to make strong claims about the precise way in which the biological brain implements this proposed mechanism. Specifically, a monosynaptic, plastic connection from hippocampal to neocortical memory engram cells may indeed be unlikely, whereas a multisynaptic pathway via the entorhinal cortex seems more plausible – but capturing it in finer detail exceeds the model's intended scope. We hope that the above addition to our manuscript will moderate the readers' expectations in this regard.

Lastly, regarding the reviewer's point about bidirectional interactions be-

tween hippocampus and neocortex – in our model, the synaptic connections between 'HIP' and 'CTX' are unidirectional, as no synapses in the reverse direction were necessary to enable our envisioned memory consolidation mechanism. That being said, one could expect such synapses to serve key roles for fear memory – e.g., in the context of re-consolidation, when the remote recall of a memory reinstates its hippocampal representation. Thus, we now explicitly mention this limitation in our discussion:

'Further, it should be noted that the 'standard theory of memory consolidation' – to which our model subscribes by positing that transient, hippocampal memory traces guide the strengthening of persistent, neocortical representations – has recently been scrutinized (cf. [7] for a detailed review). For instance, future models may reconsider our simplistic assumption that consolidation is unidirectionally driven by $HIP \rightarrow CTX$ and $HIP \rightarrow BA$ synapses. A $CTX \rightarrow HIP$ feedback mechanism could, e.g., be used to account for reconsolidation; a cognitive process relevant for exposure-based therapies [8]. In short, reconsolidation describes a process in which recalling a (cortical) long-term memory re-engages hippocampal circuits, temporarily rendering the memory modifiable before it is restabilised [9].'

R1-Maj2

The benefits of consolidation for memory retention appear limited in the current model. For example, Figure 4c shows that consolidated cortical memories decay linearly over time and would eventually be lost, suggesting that consolidation provides only a short-term advantage. This seems like an important limitation. The authors should consider discussing or implementing mechanisms that help stabilize cortical representations over longer timescales.

We thank the reviewer for bringing up this important point. It is true that, while cortical memory traces in our model decay at a much slower rate than hippocampal ones, they are not eternal. To permanently retain cortical memory traces in our model, they, too, would have to be replayed – though with less temporal urgency than hippocampal ones. This could occur via an intrinsic mechanism, as proposed for 'HIP', and/or it could be supported by external activity, for instance via replay in 'SC'.

There may also be additional mechanisms for retaining long-term memories, lying further outside the scope of our present model, such as processes at a molecular level that protect strengthened synapses from degradation (e.g., dendritic spine stabilization [10], persistent protein expression for LTP maintenance [11]). To make this limitation transparent, we have added the following paragraph to our discussion:

'Moreover, replay in the biological brain is not exclusively hippocampal, but has been found to occur independently, e.g., in the dorsal striatum [12] and vi-

sual cortex [13]. In relation to this, we should note that, while cortical memory traces in our model decay at a much slower rate than hippocampal ones, they are not eternal (cf. Figure 4c). Hence, our model would predict that cortical traces, too, need to be occasionally replayed, though with less temporal urgency, to be permanently retained. Neural replay in memory systems beyond the hippocampus is an intriguing possibility [14]. It may protect synaptic engrams from degradation in conjunction with other, *biochemical* positive feedback loops for, e.g., sustaining protein activation or synthesis [15, 11].'

R1-Maj3

Greater consistency in mechanistic assumptions across simulations would strengthen the model. Currently, some implementation details appear only in specific modules or simulations, making it unclear which assumptions apply universally. For instance, it is not clear whether the suppression of fear-inhibiting BA_I cells is learned or hard-coded, whether the higher activation threshold for BA_I versus BA_P cells holds across simulations, or whether the boost in cell recruitability applies only to the amygdala or more broadly. Clarifying which mechanisms are implemented universally and which are specific to particular simulations or model components, and specifying which results rely on these assumptions being simulation- or component-specific, would help readers better interpret the results.

Our reason for discussing certain parameter choices in the context of specific simulation results – rather than in a Methods section – is that we think this should generally make it easier for the reader to follow along. Regardless, all simulation results reported in the paper are performed using the same set of model parameters. We thank the reviewer for correctly pointing out that this is not self-explanatory. The introduction of our results section now reads:

'In the following, we demonstrate our model's behaviour across a range of simulations that target different facets of fear memory formation, consolidation, and retrieval. All simulation protocols are provided in S3 Appendix. A full list of the model's default parameter values is given in Table C of S1 Appendix. Unless stated otherwise, these values are consistent across all reported simulations.'

We agree that there are a few cases in which our manuscript should be clearer about mechanisms that only apply to certain modules. In the section on recruitability, we added:

'[Our implementation reflects the finding that fear memories formed close in time to each other show greater neural overlap.] Although this finding also holds outside the amygdala – and for memories not related to fear [16, 17] – we only apply the above recruitability mechanism to the valence-coding BA_P and BA_I regions. For simplicity, which cells form context-coding engrams in HIP, CTX and BA_N depends entirely on the features of the context presented

during memory formation.'

This simplifying assumption makes it easier for us to perform experiments probing the generalization of fear and safety across contexts with a prescribed level of similarity. The model's lack of a recruitability mechanism outside of BA is now also noted in our discussion, as per our reply to R2-Maj2.

Similarly, in connection with R1-Maj2, we added to the homeostasis section:

'In our present model, synaptic sleep homeostasis is only applied to synapses onto the valence-coding BA_P and BA_I modules, where it affects fear expression. It should be noted that biochemical mechanisms protecting specific synapses from degradation may play key roles more broadly, for instance for the retention of long-term memories [15].'

R1-Maj4

The model's ability to align with related empirical findings on fear memory consolidation remains unclear. For instance, studies have shown that hippocampal replay selectively prioritizes fear-related experiences (Wu et al., 2017), and that strong aversive events can retroactively strengthen neutral memories formed days earlier (Zaki et al., 2025). In the current model, replay and hippocampal-to-amygdala connections do not vary with valence, and valence does not modulate replay. As a result, it is not obvious whether the model could account for such findings. It would be valuable to discuss or explore potential mechanisms that might allow the model to capture these effects.

We thank the reviewer for pointing us to these empirical results. We particularly appreciate the reviewer's recommendation of discussing the apparent prioritization of emotional/aversive memories for hippocampal replay. We considered this phenomenon while developing our model – although our current implementation does not account for it, it would be relatively straightforward to implement via excitatory $BA \rightarrow HIP$ feedback during *Sleep*, or an enhancement of HIP plasticity during prediction errors in *Perception* mode. We now note in the discussion, when discussing limitations tied to our assumption of *unidirectional* $HIP \rightarrow CTX$ and $HIP \rightarrow BA$ signalling:

'Similarly, there is a range of findings hinting at computational roles for projections from the amygdala to the hippocampus [18], which our model does not account for. For instance, the hippocampus does not only encode an animal's environmental-, but also interoceptive context [19] and internal-state cues can indeed serve as conditioned stimuli for fear conditioning [20]. $BA \rightarrow HIP$ feedback may further be hypothesized to contribute to the enhanced storage of emotional memories [21], e.g., by biasing hippocampal sleep replay towards memory traces associated with harm [22].'

Here, we do not cite Wu et al. because, from our understanding, their study from 2017 refers to hippocampal replay in the context of trajectory planning rather than memory consolidation, thus lying further outside our model's scope.

Integrating memory linking effects into our model is an interesting suggestion for future work. Within our present framework, integrating these effects would be more challenging as they presumably rely on more complex replay dynamics (possibly including the co-activation of distinct hippocampal patterns, as noted by Zaki et al. [23]). We now discuss this limitation, and have added additional references to experimental findings, in our revised discussion section.

'Although coordinated hippocampus-mPFC-amygdala activity after fear learning has been reported [22, 24], temporally precise, across-region engram co-activations during sleep have not yet been demonstrated. The true dynamics of cross-regional replay are more complex than our model can account for, involving, e.g., nested or rapidly alternating ('flickering') hippocampal replay patterns during sleep [25]. Relaxing the assumption that hippocampal and amygdalar patterns are reactivated synchronously may naturally account for memory-linking effects, whereby neutral memories formed close in time to an aversive event, but in a different context, gradually acquire negative valence [23]. For instance, this may hold if hippocampal 'flickering' could cause a BA ensemble to co-activate with distinct HIP engrams in quick succession. Memory linking may also relate to an increased neuronal overlap between hippocampal memories formed close in time [17, 26, 27], which could be explained by a neuron-specific mechanism similar to our model's '$BA_{P/I}$ cell recruitability'.'

2.2 Minor comments [R1-Min]:

R1-Min1

The claim "Ours is the first to incorporate a neural mechanism enabling" fear memory consolidation could be softened, given that models such as Mattar & Daw (2018) also simulated consolidation of fear memory. A more accurate phrasing might be that this is the first model to implement systems consolidation of fear memory.

We thank the reviewer for pointing us to the paper by Mattar & Daw, which is very relevant. Although their proposal is not strictly a neural network model, we agree that our phrasing should have been more careful. We have now opted for a more neutral phrasing:

'Our model integrates mechanisms of fear learning, systems consolidation and synaptic homeostasis to provide a unified account of how contextual fear memories form and evolve over time.'

R1-Min2

In Figure 1 (page 5), the legend refers to "Nodes labelled with an S-shape". I am not sure which nodes are marked that way.
We meant to refer to the sigmoid symbol marking the 'CeA'- and 'U'-nodes as an 'S-shape', which may have accidentally made things *less* clear. The legend now refers to

'Nodes labelled with a sigmoid'.

R1-Min3

On page 9, "In the following, we . . . " seems incomplete.

Please see our response to R1-Maj3 for the updated paragraph.

R1-Min4

Also on page 9, please add a space in "inRecall mode" so that it reads "in Recall mode."

Thank you, we have corrected this.

R1-Min5

In Figure 5c (page 13), what does the y-axis label "CeM" refer to?

We apologize for this oversight. In an earlier version of our draft we had referred to 'CeA' – our model's fear output node – as 'CeM': the medial subdivision of CeA (see, e.g., [28]).

R1-Min6

When discussing insomnia and sleep disruptions (page 14), the motivation would be clearer if these pathologies were tied more directly to fear acquisition and expression.

We have extended the introduction to the relevant section, aiming to demonstrate our motivation for investigating potential effects of sleep omission in our model more clearly:

'Insomnia—a chronic difficulty in falling or staying asleep—is common across anxiety disorders [29, 30]. In the aftermath of traumatic events, sleep disruption is a strong predictor of later PTSD, though causal links have remained

unclear [31, 32]. Fear conditioning studies have pointed at ways in which sleep omission may exacerbate fear memories. For instance, sleep between sessions has been shown to counteract sensitization to experimental stressors [31, 33, 34], and sleep deprivation may enhance fear expression in both rats [35] and humans [36, 37]. In contrast, other studies have proposed deliberately *avoiding* sleep in the aftermath of trauma, as a means of interfering with memory consolidation and preventing later PTSD [38, 39]. Clarifying the involved neural mechanisms will be key for reconciling these lines of evidence.'

R1-Min7

The Kitamura et al. study is described in detail in the Discussion, where readers might expect a higher-level summary. The authors might consider introducing it earlier to help motivate the model's architecture, or alternatively, making the description in the Discussion more concise.

Indeed, the study by Kitamura et al. played an important role for the design of our model., since the authors explicitly investigated engram cells in hippocampus, neocortex and amygdala at different time points following contextual fear conditioning. However, we agree that readers would expect our discussion to position these findings in the context of other studies in a more compact way. We have revised our discussion with this rationale in mind, including the following excerpt:

'Consistent with the standard model of systems consolidation [40, 41], hippocampal engrams become functionally silent within two weeks [4, 42]. In contrast, the mPFC engram remains silent while the memory is recent but matures in the weeks following its formation, eventually supporting remote memory recall [4, 43, 44, 45]. This strengthening of cortical synapses relies on sustained hippocampal inputs [4, 46], supported, e.g., by hippocampal sharp-wave ripple (SPW-R) events that primarily occur at rest [47, 48, 49]. Experimental manipulations indicate that mPFC-to-BLA projections become essential for memory expression approximately two weeks after CFC in rats and, notably, that the implicated BLA cells strongly overlap those previously targeted by HIP [4].'

We continue to refer to the study by Kitamura et al. at several points throughout our manuscript, first at the onset of our model description:

'[...] Building on these proposals, we provide a computational account of how context fear memories transition from transient hippocampal-amygdalar representations into stable amygdalo-cortical associations [4].'

3 Reviewer 2

3.1 Major comments [R2-Maj]:

R2-Maj1

This work focuses mainly on how fear memory acquisition can be enhanced and generalised. This is valuable, as it helps to elucidate potential neural mechanisms underlying these phenomena through computational modelling. However, what is perhaps more interesting—and more relevant for translational neuroscience—is how established fear memories can be weakened, particularly traumatic memories. I would like to see some simulations targeting this point included in the paper. There is a short section on fear extinction, but it is too brief and lacks sufficient detail on how sleep, de-stressing, or other potential factors may lead to fear memory weakening/extinction. For example: • Line 334: "... enhancing synaptic sleep homeostasis in the amygdala could be a key therapeutic strategy in PTSD and related disorders." I would expect the authors to demonstrate this directly in their model. • Figure 7: the authors show that chronic sleep deprivation leads to accelerated fear acquisition. I would also expect to see conditions that decelerate fear acquisition.

We thank the reviewer for this detailed comment. We agree that improving approaches towards treating pathological (e.g., trauma-related) fear, in particular, is a key motivation behind studying the neural mechanisms of fear memory. Indeed, our main motivation for focusing our model around memory *consolidation* was to move towards a discussion of *chronic* fear symptoms in PTSD, which often emerge over longer time frames. Within our Lab, we are currently working on a model extension that will allow us to investigate traumatic memories, using the work presented in this manuscript as a starting point.

However, in the present submission, we do not yet provide simulations that specifically target the pathophysiology of PTSD. The enduring intensity of traumatic memories in the disorder may indicate that memory representations are abnormally persistent in their original form (e.g., [50, 51]). It is intriguing to hypothesize links between this common assumption and a failure of the consolidation mechanism sketched by our model. On the other hand, the sensory vividness and (often) dissociative nature of traumatic memories in PTSD indicate that they may not be fully described by the re-activation of a context-coding cell ensemble [52]. Therefore, investigating traumatic memories may require characterising the ways in which they diverge from the consolidation framework proposed here. While we believe that this is a very worthwhile task, which may allow us to propose qualitative differences between traumatic memories and 'normal' contextual fear memories, it falls outside the intended scope of our current manuscript.

Further, we agree with the reviewer that it is helpful for us to elaborate

on the implications of our model's extinction mechanism. Fear extinction is the cognitive process underlying exposure therapy. Our previous submission already contained simulation results on *fear renewal* – a return of conditioned fear after moving away from the extinction context. This phenomenon – and *ABC* renewal in particular [53] – is highly relevant to exposure therapy, since fear extinguished in the clinic often returns during later experience. Therefore, it is highly relevant to ask whether there are strategies that limit the return of fear in this paradigm. In the behavioural literature, conducting fear extinction in multiple contexts has been proposed as a promising avenue (e.g., [54, 55]), though conflicting experimental results exist (e.g., [56, 57]).

Therefore, our revised manuscript includes a simulation probing the circumstances under which distributing fear extinction across multiple contexts provides a clear benefit in our framework. In short, our model predicts that, when a fear association is strong enough to generalize even to quite dissimilar contexts, extinction training in the conditioning environment is unlikely to prevent fear renewal in *all* of those contexts. On the other hand, distributing fear extinction across a range of moderately similar environments resulted in more generalized safety memories in our simulations. Our framework thus suggests that *aiming for the largest possible similarity between extinction and acquisition context* may not always be an ideal strategy for exposure therapy.

Below, Fig 1a shows the average evolution of our model's fear response in two related simulation protocols. The upper plot depicts *ABC* renewal; fear is acquired in context A, extinguished in a similar context B, but returns upon exposure to another similar context C. In the lower plot, extinction is instead distributed across four contexts B_1 to B_4. In these simulations, fear expression remained higher over the course of the extinction phase. However, the repeated re-emergence of positive prediction errors allowed a more robust strengthening of $BA_N \rightarrow BA_I$ synapses, which caused the model's fear response to be inhibited when, ultimately, context C was presented.

Fig 1b illustrates the average magnitude of fear renewal in context C, depending on the similarity (*input overlap*) between acquisition context A and the extinction contexts $B_1, \cdots, B_4$, for several levels of the overlap $A \leftrightarrow C$. In our simulations, distributing extinction across multiple contexts generally lowers fear renewal in C. For renewal contexts very similar to A, using a set of B_i that share a large overlap with A is most effective. However, as the similarity between A and C decreases, introducing greater differences between A and the B_i becomes beneficial, since additional variation in the B_i allows extinction to generalize more broadly.

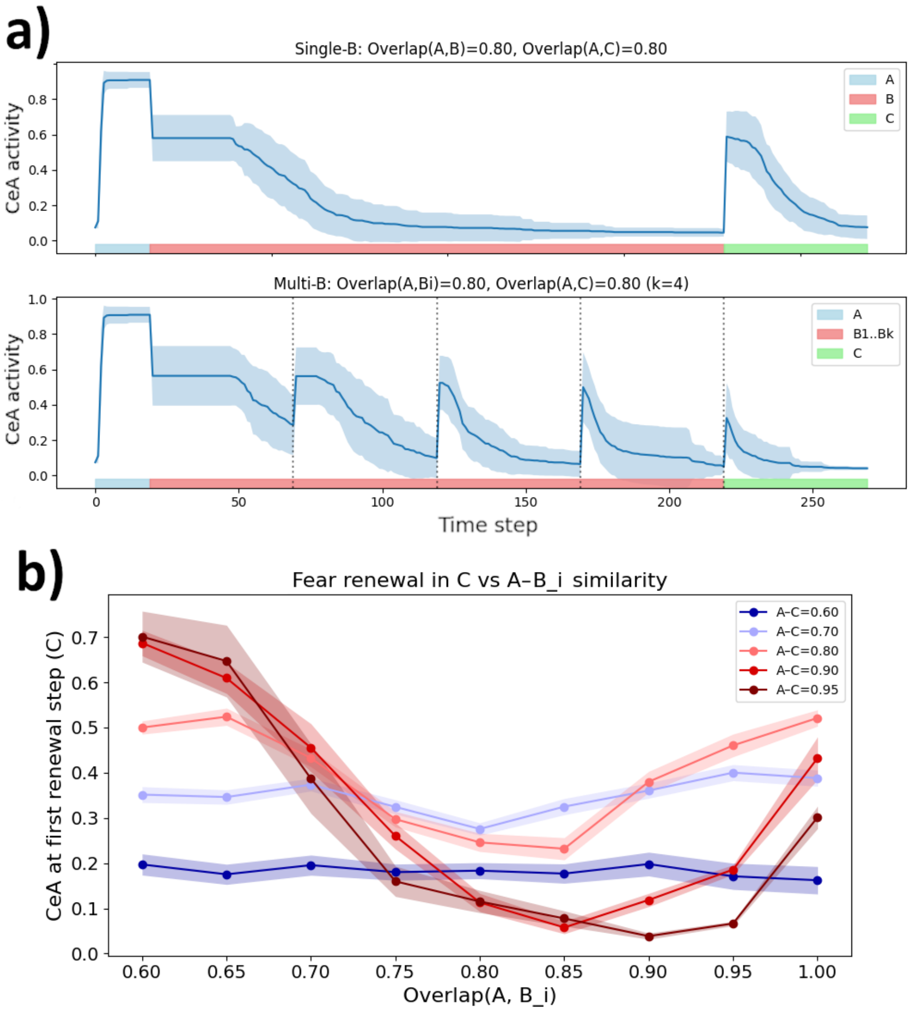

Figure 1: **ABC Renewal with extinction in multiple contexts.**
Please see our revised manuscript for the full section on multi-context extinction.

Regarding the reviewer's comment on the effects of sleep on fear memory weakening, our model's homeostasis rule is designed in a way that generally decreases the overall synaptic density in the fear-coding $BA_N \rightarrow BA_P$ pathway. A straightforward corollary of our results on chronic sleep disruption is that *prolonging* sleep will generally slow down future fear acquisition (and reduce fear generalization) – assuming that synaptic homeostasis occurs at a stable rate throughout sleep. Increasing the rate at which these homeostatic changes occur will likewise *lower* the model's tendency towards fear acquisition. On the other hand, when we alter the form of the homeostasis rule – making the synaptic pruning less aggressive, and instead favouring the retention/consolidation of $BA_N \rightarrow BA_P$ synapses – longer sleep durations no longer de-sensitize the model. We now highlight these observations with the help of additional simulations in our Supplementary Materials (cf. Figure 2). In the section on sleep deprivation, we summarize these simulations as follows:

'To illustrate that *Sleep*'s impact on future fear acquisition is mediated by our model's homeostasis mechanism, we repeated the above procedure for several variants of our model, with altered parameters (cf. Supplementary Fig S5). When we increased (decreased) the rate of homeostatic plasticity on $BA_N \rightarrow BA_P$ synapses, fear acquisition was weakened (strengthened) for all *Sleep* durations, without disrupting the monotonic relationship between *Sleep* length and fear sensitization. On the other hand, when we lowered the minimum strength a $BA_N \rightarrow BA_P$ synapse must have to be consolidated rather than pruned by the homeostasis mechanism (the *extinction threshold*, cf. Fig 3b), extended *Sleep* durations enhanced acquisition – relative to the baseline model and to shorter *Sleep* durations, though not relative to a complete lack of *Sleep*. This indicates that *Sleep*'s de-sensitizing effect on fear acquisition relies on intact synaptic pruning.'

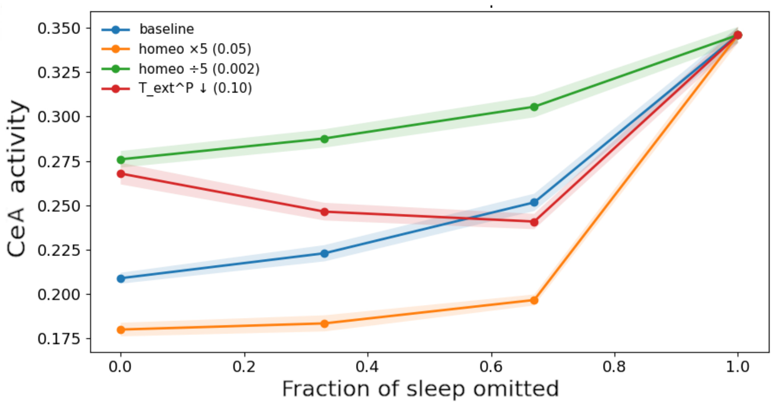

Figure 2: **Effects of sleep deprivation for different model variants.**
As discussed in the document for Supplementary Figure S5, different variants of our model, with different daily amounts of *Sleep*, were subjected to 7 days filled with exposure to various contexts, before being briefly tested for fear acquisition in a novel environment. The models' CeA output was recorded after 3 time steps of US delivery (strength 0.6).
Dots denote the mean CeA output, shaded regions one standard error across 200 runs of the simulation.
For the default model configuration ('baseline'), as well as for model variants with accelerated ('homeo ×5') or decelerated ('homeo ÷5') homeostatic plasticity, fear acquisition scaled inversely with *Sleep* duration.
For the variant with a lowered *extinction threshold* of the homeostasis rule acting on $BA_N \rightarrow BA_P$ synapses ('T_ext^P'), extended *Sleep* durations no longer had this moderating effect.
Note that, when 100% of *Sleep* were omitted, the different model instances behaved identically, because we used random seeds to prevent any stochastic differences in terms of context generation, weight initialization or cell recruitability

R2-Maj2

I have concerns about the modelling of hippocampal replay. From Ji & Wilson, we know that replay coordinates sequential activity between the hippocampus and visual cortex, but this occurs during specific windows of sleep. In the current context, "replay" is used to establish communication between hippocampus, cortex, and amygdala. This does not align precisely with the timescale of replay, which is usually tens to hundreds of milliseconds. For example, it is unclear what the unit of the time step in Fig. 4b is. In the text you mention "over the course of a sleep phase", but it is not clear what duration you mean by a sleep phase. Is it an entire NREM episode? At other times the timescale is given as "days" (e.g., Fig. 5d and Fig. 8d). The authors need to make the timescale consistent, or, if that is not possible, provide clearer descriptions.

We appreciate the reviewer's feedback regarding the timescale of replay. Our implementation of hippocampal replay (as well as the parameters guiding sleep duration, convergence and inhibitory 'cell adaptation' – and thus the rate of replay) was adopted from a model by Fiebig & Lansner (2014) [5]. There, the authors refer to a time step length of 10ms, such that replay events had a duration of tens to hundreds of milliseconds. They noted:

'As we undertake a full simulation cycle of one day and one night in just 330 time steps (Figure 4) of 10 ms each, the model plasticity is scaled against reality by a factor of roughly 26.000. The chief motivation for this is to enable a study of systems of this kind at all. (...) Note that this is a scaling of plasticity only and does not include a scaling of the neural dynamics.'

We respect the authors' attempt to explicitly specify the timescale of their model. However, the actual temporal dynamics of hippocampal replay are far more complex (e.g., with special roles played by fast population bursts) than their (and, by extension, our-) model is able to capture. Therefore, we decided to leave the time step duration in our manuscript indeterminate. We now mention this level of abstraction in our discussion section, as well as when presenting our initial results on sleep replay (see further below):

'Clarifying the temporal organization of cross-regional replay and the synaptic mechanisms underlying systems consolidation remain key goals for future experiments. Regarding timescales, our replay implementation after Fiebig & Lansner (2014) assumes that several recently formed hippocampal memories are reactivated within a 'night' [5] – with each replay event lasting several simulation steps, but leaving the time step duration indeterminate. This is a functional abstraction; we do not model the oscillatory dynamics that may orchestrate these replay events (e.g., sharp-wave ripples [22]), nor the temporal arrangement of hundreds of these events over the course of REM [58] and slow-wave sleep [59].'

Further, we apologize for our previously ambiguous use of the term 'sleep phase'. Here, we intended to refer to time windows spent by our model in *Sleep*, as opposed to *Perception* or *Recall*, mode. Where applicable, we have revised our phrasing to be clearer in this regard.

Lastly, we would like to clarify that, in the simulation protocols and figures referring to a timescale of 'days', a 'day' does *not* correspond to a time step – but to a block of time steps spent in *Perception* and/or *Recall* mode. Here, different 'days' are interspersed with instances of sleep, each lasting 165 time steps (except in Figure 7, where that duration is altered). We now state this explicitly when presenting the first long-timescale simulation of our manuscript.

'In our simulations, the default duration of *Sleep* is 165 time steps. As a functional abstraction, we leave the duration of an individual time step indeterminate. In reality, hippocampal replay is primarily observed during sharp-wave ripple events, which last tens to hundreds of milliseconds and occur dispersed throughout sleep [60, 61]. In the following simulations, *days* refer to time win-

dows the model spends in *Perception* and/or *Recall* mode (one or several experimental sessions). Days are interleaved with blocks of *Sleep*.'

R2-Maj3

I ran the authors' provided code and was able to reproduce the figures in the main text. This is a relatively complex model with many parameters, and I give credit to the authors for making this model work as intended. However, I am somewhat concerned about the robustness of the reported results. For example, if a parameter setting were altered, would the results remain qualitatively similar? Or is the current configuration "cherry-picked"? A stability analysis of the model would strengthen confidence in the results.

We thank the reviewer for taking their time to run the provided code, and we thank them for their positive comments. We agree that a stability analysis is useful for making our simulation results more transparent. In our supplementary materials, we have now evaluated the robustness of some of our key results under perturbations of certain parameters. Our overall approach was as follows:

For any simulation result that we chose to evaluate, we started by selecting a set of model parameters that we expected to affect the model mechanism under investigation (e.g., fear generalization, fear sensitization after sleep disruptions) the most. After defining suitable parameter ranges, we collectively resampled the identified parameters N times, altering, e.g., the model's sensitivity to US signals or parameters of the homeostasis rule acting on $\mathrm{BA_N} \rightarrow \mathrm{BA_P}$ synapses. For each parameter set, we instantiated the model and ran the given simulation protocol, collecting relevant output measures (e.g., fear expression in a range of 'similarity contexts' when evaluating the results in Fig 5b). Each configuration was repeated K times, using different 'noise seeds' (affecting, e.g., the *recruitability* of $BA_{P/I}$ cells, weight initialization, and context generation). After averaging the given output measures across those K runs, we evaluated several pass/fail criteria, capturing the qualitative results we highlighted in the relevant part of the main text.

Most, though not all, hypotheses evaluated in our robustness analysis could be reproduced by a majority of the randomly sampled parameter sets. For the tests that had a pass rate below 50%, we included additional figures providing more clarity on the parameters that affected their success rate or, in one case, indicating that the relevant result at least held in the average *across parameter sets*. Further explanations for why certain tests achieved a lower pass rate are provided below.

Arriving at a parameter configuration that cleanly displays *all* behaviours reported in our main manuscript did require some care, but we hope that our analyses can strengthen confidence that the shown outcomes do not depend on a single specific, 'cherry-picked' parameter combination. In the following, we

summarize the outcomes of our analyses, which are detailed in S3 Appendix:

1. As long as BA_I cells have higher firing thresholds and slower learning rates than BA_P cells, making it so the latter tend to be recruited into a context with a 'larger margin of safety' in terms of synaptic strengths, fear extinction is *generally* more context-specific than fear acquisition. 540 out of 600 parameter sets recovered the finding that, after fear acquisition and extinction in the same context, a return of fear specifically occurs in *moderately* similar environments. This matches Fig 4d of our manuscript.

2. Based on the simulation protocol used to investigate increases in fear generalization (Fig 5d of our draft), fear conditioned in a context 'A' was successfully recalled on each of the next 15 days for 19 of 50 parameter samples. We identified the firing threshold of $\mathrm{BA_P}$ cells and the *extinction threshold* of the $\mathrm{BA_N}\to\mathrm{BA_P}$ homeostasis rule as the most critical parameters in this regard; if their values are too high, fear associations are at increased risk of being forgotten. In our main results, we further report that a harmless context B, sharing a 60% input overlap with A, does not spark fear early after conditioning, but does so after systems consolidation of the involved memory traces. This specific outcome was replicated by just 6 of the 50 parameter sets. It is not surprising that this result is more delicate since it depends on successful retention of the fear memory, as well as on quantitative assumptions about the fear generalization gradient both before and after consolidation. Our main simulation results do indicate that the time course of increases in fear expression in B matches precisely that of the switch from hippocampal to cortical recall, so that we remain confident in the reported model mechanism.

3. Based on the simulation protocol from our section on sleep deprivation (Fig 8 of our draft), we tested the hypotheses that the net synaptic strength of $\mathrm{BA_P}\to\mathrm{BA_N}$ synapses, as well as fear acquired in a novel context, are *strictly decreasing* functions of the model's daily *Sleep* duration. The first hypothesis held for all 50 parameter sets. The assumption that sleep promotes an overall weakening of synaptic strengths (cf. Tononi et al. [62, 63]) is built into the form of our homeostasis rule, fairly independently of its parameters. On the other hand, fear acquired in a novel context was strictly decreasing for only 16 of the 50 parameter samples. In part, this lower pass rate is explained by the fact that fear acquisition in our model is relatively noisy, depending on current $\mathrm{BA_P}$ cell recruitability, the form of the conditioned context, as well as pre-existing synaptic weights. Moreover, if the parameters of the homeostasis rule are altered in a way that makes moderately weak $\mathrm{BA_P} \to \mathrm{BA_N}$ synapses more likely to be preserved rather than pruned overnight, extended *Sleep* durations do indeed no longer have a weakening effect on future fear acquisition (cf. Fig 2).

4. In our section on Stress-Enhanced Fear Learning, we incorporated neural effects of psychological stress into our model. The presentation of extreme

US signals activates a transient increase in BA_P recruitability, as well as a temporary change to *Sleep* homeostasis that limits the overnight pruning of $BA_N \to BA_P$ synapses. Here we evaluated the hypothesis that, 15 days after activating these stress effects, our model has a higher $BA_N \to BA_P$ net synaptic weight and acquires fear more rapidly in a novel context, relative to an 'unstressed' control model (Fig 9 of our draft). A higher synaptic weight was observed for all 50 parameter samples. Further, in the average across five noise seeds, the previously stressed model acquired more fear in the novel context, relative to the control, for 44 out of 50 parameter sets. Hence, the transient stress effects in our model produce robust increases in fear acquisition, long after the affected parameters have returned to their baseline.

We believe that these analyses re-iterate our most important simulation results. Several simulation protocols were not evaluated in this way. For instance, we did not assess ABA, AAB and ABC renewal, since these findings follow logically from the context specificity of fear extinction. Nor did we evaluate Stress-Enhanced Fear Learning *without* delay after a 'traumatic' stressor, since it follows trivially from the temporary increase in BA_P cell recruitability which occurs after our model receives an extreme US signal. For more details on all stability analyses, including lists of all parameters that we resampled, please see S3 Appendix of our revised manuscript.

R2-Maj4

Although the model explains previous empirical findings, makes testable predictions, and links these results to potential neural mechanisms, there remains a significant gap between the computational assumptions/mechanisms and the actual neural mechanisms underlying these phenomena. For example: • Line 314: "Our model can explain these findings due to our assumption that sleep after fear learning prunes weak synapses onto P-cells." • Line 331: "Targeting sleep disruptions ... may directly influence the synaptic mechanisms underlying chronic anxiety." • Line 383: "... implies that therapeutic interventions targeting either sleep quality or resilience to stress-induced homeostatic dysregulation could reduce vulnerability to pathological fear and anxiety disorders." These claims are somewhat too strong, given that they derive from a computational model rather than direct empirical evidence. The language could be moderated to avoid potentially misleading over-interpretation. I understand that some of these issues are difficult to address fully in a computational modelling study. Nonetheless, I find the model valuable.

We agree with the reviewer that our wording should be careful not to encourage over-interpretation of our results. There is a fine balance to strike between highlighting that our modelling assumptions have real implications for the investigated neural mechanisms, and acknowledging that our work is intended to guide experiments rather than serve as conclusive evidence. We have revised

the manuscript with this rationale in mind. For the specific excerpts mentioned by the reviewer, we have made the following changes:

- 'Line 314: In our model, sleep offers protection from fear sensitization – exaggerated fear learning and expression – by driving a regular pruning of weak $BA_N \rightarrow BA_P$ synapses.'
- 'Line 331: In summary, our model predicts that disrupting sleep – especially after aversive experiences – is likely to produce net increases in amygdalar synapse density, promoting fear expression and accelerating future acquisition. This suggestion highlights synaptic sleep homeostasis as a potential mechanism linking sleep disruptions to anxiety symptoms in relevant disorders, such as PTSD [64, 65].'
- Line 383: 'Clinically, this hypothesis – if validated – would suggest that therapeutic or pharmaceutical interventions targeting either sleep quality or resilience to stress-induced homeostatic dysregulation could reduce...'

3.2 Minor comments [R2-Min]:

R2-Min1

Line 22: "... also exhibits activity coordinated with hippocampal replay during sleep... " — needs citations.

Thank you for spotting that the citation was missing here, Girardeau et al. (2017) is the key source for this claim [22]. Previous studies had shown coherent activity between hippocampus and amygdala in the theta range during REM sleep – a finding that does not directly indicate neural replay, but that has been hypothesized to be linked with emotional memory [66].

R2-Min2

Is there empirical evidence supporting the choice to model CTX as a BPCNN and HPC as a WTA network? How do these differ from a Hopfield network, and why was a Hopfield network not used instead?

The choice of modelling CTX as a BCPNN and HIP as a WTA network was adopted from Fiebig and Lansner [5], who proposed a model of systems consolidation driven by endogenous, overnight hippocampal replay. We used this paper as the starting point for our model, asking how to integrate associative fear memories into this existing framework, and this is the practical reason why we did not consider Hopfield networks for our implementation. Yet, there are also theoretical reasons for why a BCPNN may be preferred for our task, which we will touch on in the following.

The neural architecture of BCPNNs is inspired by the columnar organization of the cortex [67]. Mathematically, the learning rule used in our implementation, as per Sandberg & Lansner [68], allows the network to infer correlations ($\propto$ synaptic weights) between variable values ($\equiv$ units). The computational units are organized into cortical 'hypercolumns' which, in the mathematical derivation of the model, correspond to distinct variables. In a BCPNN, unit activities within each hypercolumn (corresponding to one variable) are normalized so that they sum to 1 – this is argued to correspond to competitive neuronal competition within hypercolumns. Presumably, one key reason Fiebig & Lansner used a WTA rule to compute neural activities within the HIP module is that the concept of hypercolumns does not apply to the hippocampus. Further, memory engrams in the hippocampal dentate gyrus (DG) and CA3 are found to be sparse (with, e.g., a remarkably consistent fraction of $2-6\%$ active cells in DG) – which does not match the aforementioned activity normalization performed in BCPNNs. The 'hard WTA' constraint enforced on HIP activity in our model should – abstractly – be taken to correspond to inhibitory microcircuit mechanisms that are assumed to enforce engram sparsity in the biological hippocampus [69].

BCPNNs and Hopfield networks are conceptually similar, in the sense that both are associative memory systems allowing for point attractor dynamics. However, Hopfield networks are usually trained by presenting a set of memory patterns which are then stored, not taking into account how recently a pattern was observed. In contrast, with the given learning rule, BCPNNs (and the WTA networks derived from them) store stronger representations for contexts that have recently been presented or replayed. On the one hand, this is important for HIP's ability to independently replay memories that have recently been formed – i.e., those that still need to be consolidated – without requiring an additional tagging mechanism. On the other hand, since new patterns are learned gradually, implicitly overwriting previous learning step-by-step, older memories are forgotten more 'gracefully' than in a Hopfield network which – as noted by the reviewer in R2-Min4 – can be prone to the 'catastrophic' forgetting of a large fraction of memories when their storage capacity is surpassed.

We provide a motivation for using BCPNN and WTA networks for our CTX HIP/BA_N modules, respectively, in Appendix S1 of our revised manuscript.

R2-Min3

In the author summary, the authors ask: "Why do some fear memories fade while others persist or even grow stronger over time?" After reading the paper, I did not find a clear answer. Can the model explain this? (This relates to my major comment 1.)

Identifying the neural mechanisms that determine which memories the brain retains, as well as those that affect its associated valence in the long run, are key challenges. Providing pointers on this subject is one of the main motivations

behind our model. We have revised the final conclusion of our manuscript to touch on this point more explicitly:

'[...] Our model suggests that the maturation of a cortical–amygdalar engram for the remote recall of contextual fear memories [4] relies on simultaneous co-activation of cortical and amygdalar engram cells, driven by hippocampal replay events during sleep. The model further predicts that the long-term evolution of an aversive memory depends on whether the involved amygdalar synapses are stabilised or pruned during offline memory consolidation. We hypothesize that a stress-induced failure of synaptic pruning in the aftermath of a fear learning event favours the accumulation of synaptic strengths in amygdala circuits and may thereby contribute to a chronic fear-sensitised state.'

R2-Min4

Line 76: the authors state that representations in the hippocampus are short-lived and overwritten by new learning. In Hopfield-like models, this would be termed catastrophic forgetting. Why is this not the case for the neocortex in the proposed model?

Catastrophic forgetting refers to the abrupt loss of previously stored memories in a network as new learning occurs. BCPNNs, using the learning rule we adopted from Fiebig & Lansner [5], implicitly overwrite older memories gradually. In other words, individual stored representations tend to continuously grow weaker as they age, until they eventually become inaccessible. This gradual loss of recallable information is distinct from 'catastrophic' forgetting, which involves the sudden loss of (sets of memories) when a network's storage capacity is exceeded – this was described in greater detail by Sandberg and Lansner (2002; [68]), who introduced the given learning rule.

That being said, it is true that cortical memory traces in our model are also prone to being eventually forgotten if they are not refreshed for a sufficiently long amount of time. To represent life-long memories, an extension of our model may, e.g., use a replay mechanism for cortical memories, potentially operating on a slower time scale than what is the case for the hippocampus. In the present revision, we note this limitation in the discussion, as well as in the context of Figure 4c. For more on this, please see our response to R1-Maj2.

R2-Min5

Line 79–80: replay of sequential patterns is implemented via inhibitory synapses operating at a fast timescale. Previous works (e.g., Azizi et al., 2013; Ji et al., 2024) used adaptation to generate replay-like dynamics. What is the difference between these approaches, given that the present model does include adaptation (Eq. 7–10)?

We thank the reviewer for pointing us to these previous works. Our model's use of a 'Hebbian depression' mechanism, preventing HIP from resting in the same attractor state and thus allowing it to cycle through different memory patterns, is adopted from the model on systems consolidation by Fiebig & Lansner that served as the starting point for our implementation [5]. This mechanism was first integrated into a BCPNN by Sandberg & Lansner [68], who proposed that it – abstractly – corresponds to a fast depression of synapses between co-activated engram cells during replay, 'alone or in combination with neural adaptation'.

Azizi et al. (2013) implemented a cell-wise 'adaptation current' in their spiking neural network model of hippocampal preplay, with the same intention of preventing the network from permanently resting in an attractor state [70]. This concept is subtly different from our 'Hebbian depression', in that it explicitly discourages the persistent firing of single units, rather than sets of co-active units. This may give rise to slightly different dynamics / state space trajectories but, qualitatively, the behaviour should remain similar. In our implementation, too, individual units are unlikely to remain persistently active across longer time windows as they receive increasingly stronger inhibition from co-active cells.

Ji et al. recently (2024) investigated the effects of modulating a cell adaptation mechanism on replay dynamics in a spiking model of hippocampal place cells, in great detail [71]. Their implementation is closer to the model by Azizi et al. than to our formulation – in the sense that it involves neural self-inhibition rather than inhibition between units – but their results resonate with several observations we made in our own simulations (not shown in the manuscript): Firstly, that the rate of cell adaptation directly relates to the speed of state space trajectories during replay. And, secondly, that if adaptation strength is increased, replay can become 'super-diffusive' which, in our experiments, often manifested as a back-and-forth between two separate attractor states.

To conclude, although the Hebbian depression mechanism used in our model differs in form from the previous works referred to by the reviewer, there are clear conceptual similarities. We have updated the corresponding part of the model description to be clearer:

'During *Sleep*, in the absence of external inputs, recurrent excitation causes HIP to converge to one of its attractor states – 'replaying' that engram. To prevent the indefinite replay of a single memory, inhibitory synapses between active cells are then rapidly strengthened via a Hebbian learning rule [5]. Eventually, HIP's activity is pushed away, allowing convergence to another attractor. This 'Hebbian depression', or 'adaptation projection' after Fiebig & Lansner [5], has been proposed to abstractly represent a combination of spike frequency adaptation and synaptic short-term depression [68]. Similar to past computational works on neural replay [70, 71], HIP in our model thus relies on a combination of excitatory attractor dynamics and a self-inhibitory fatigue mechanism to cycle through different memory patterns [72].'

R2-Min6

Line 239: "...fear extinction depends primarily on silencing, rather than erasure..." The model shows that silencing can work, but why not erasure? This was not tested.

We agree with the reviewer that our simulations do not assess whether erasing fear associations can or cannot work, and we did not intend to suggest this. The statement referred to here was meant to indicate that our way of implementing fear extinction was motivated by the finding that the brain (initially) extinguishes fear by silencing, rather than erasing the relevant association. We have updated the relevant segment:

'Our choice of modelling fear extinction through the recruitment of fear-inhibiting neurons, downstream of the encoding for the fear association, was informed by neurobiological findings [73, 74] and the observation that – at least during early stages of extinction [75, 76] – the brain primarily silences learned fear associations but initially does not erase them. Phenomenologically, this is also evidenced by the spontaneous return of previously extinguished fear, as well as context-dependent fear renewal effects – which our model reproduces, as outlined in the following section.'

Indeed, there is neurobiological evidence indicating that the brain *can* use an erasure mechanism during late stages of successful extinction learning, after repeated, safe exposure to a feared stimulus [75]. We did consider implementing such a concept in our model by introducing long-term depression of synapses onto BA_P cells that have repeatedly been inhibited by BA_I cells. This would have yielded the interesting prediction that AAB renewal – a return of fear upon encountering a context (B) similar to one in which fear has been acquired and extinguished (A) – should take place when extinction has recently taken place, but should no longer do so after repeated re-exposure to A. However, we chose not to explore this direction more deeply.

R2-Min7

Fig. 4a: why does the hippocampus rapidly form context engrams? Is this due to fast synapses?

Yes, fast Hebbian plasticity acting on its recurrent excitatory synapses, as argued to occur in CA3 in particular (e.g., [77]).

R2-Min8

Fig. 4d: why is the x-axis labelled "trials" rather than "steps"?
We thank the reviewer for pointing this out. The updated Figures 4d and 5c

now appropriately refer to 'time steps'.

R2-Min9

Line 300: what does "unchecked" mean here?

Here, the term is meant to refer to a scenario where, if the same fear-coding cells were consistently (without restriction, 'unchecked') recruited in a number of similar environments – due to a bias resulting from previous conditioning events – individual $BA_N \rightarrow BA_P$ synapses could become so strong that, soon, the activity of those postsynaptic BA_P cells to generalize to any novel environment. We have revised the statement to more explicitly say this:

'Even if the most strongly innervated P-cells initially remained below activation threshold, their subthreshold excitation would bias the model towards recruiting the same cells again to rapidly acquire fear in context 'B', should a mild aversive event occur. If this *consistently* occurred in several environments, this cascading bias would soon cause the activity of the affected P-cells to generalize to a very broad range of contexts.'

References

[1] Winocur G, Moscovitch M. Memory transformation and systems consolidation. Journal of the International Neuropsychological Society. 2011;17(5):766-80.

[2] Moscovitch M, Cabeza R, Winocur G, Nadel L. Episodic memory and beyond: the hippocampus and neocortex in transformation. Annual review of psychology. 2016;67(1):105-34.

[3] Tonegawa S, Morrissey MD, Kitamura T. The role of engram cells in the systems consolidation of memory. Nature Reviews Neuroscience. 2018;19(8):485-98.

[4] Kitamura T, Ogawa SK, Roy DS, Okuyama T, Morrissey MD, Smith LM, et al. Engrams and circuits crucial for systems consolidation of a memory. Science. 2017;356(6333):73-8.

[5] Fiebig F, Lansner A. Memory consolidation from seconds to weeks: a three-stage neural network model with autonomous reinstatement dynamics. Frontiers in computational neuroscience. 2014;8:64.

[6] Krasne FB, Cushman JD, Fanselow MS. A Bayesian context fear learning algorithm/automaton. Frontiers in behavioral neuroscience. 2015;9:112.

[7] Moscovitch M, Gilboa A. Has the concept of systems consolidation outlived its usefulness? Identification and evaluation of premises underlying systems consolidation. Faculty reviews. 2022;11:33.

[8] Vermes JS, Ayres R, Goes AS, Del Real N, Araujo AC, Schiller D, et al. Targeting the reconsolidation of traumatic memories with a brief 2-session imaginal exposure intervention in post-traumatic stress disorder. Journal of Affective Disorders. 2020;276:487-94.

[9] McKenzie S, Eichenbaum H. Consolidation and reconsolidation: two lives of memories? Neuron. 2011;71(2):224-33.

[10] Basu S, Lamprecht R. The role of actin cytoskeleton in dendritic spines in the maintenance of long-term memory. Frontiers in molecular neuroscience. 2018;11:143.

[11] Hsieh C, Tsokas P, Grau-Perales A, Lesburguères E, Bukai J, Khanna K, et al. Persistent increases of PKMζ in memory-activated neurons trace LTP maintenance during spatial long-term memory storage. European Journal of Neuroscience. 2021;54(8):6795-814.

[12] Thompson EJ, Rollik L, Waked B, Mills G, Kaur J, Geva B, et al. Replay of procedural experience is independent of the hippocampus. bioRxiv. 2024:2024-06.

[13] Wittkuhn L, Krippner LM, Koch C, Schuck NW. Replay in the human visual cortex during brief task pauses is linked to implicit learning of successor representations. Proceedings of the National Academy of Sciences. 2025;122(34):e2507516122.

[14] O'Neill J, Boccara CN, Stella F, Schönenberger P, Csicsvari J. Superficial layers of the medial entorhinal cortex replay independently of the hippocampus. Science. 2017;355(6321):184-8.

[15] Smolen P, Baxter DA, Byrne JH. How can memories last for days, years, or a lifetime? Proposed mechanisms for maintaining synaptic potentiation and memory. Learning & Memory. 2019;26(5):133-50.

[16] Sehgal M, Zhou M, Lavi A, Huang S, Zhou Y, Silva A. Memory allocation mechanisms underlie memory linking across time. Neurobiology of Learning and Memory. 2018;153:21-5.

[17] Delamare G, Tomé DF, Clopath C. Intrinsic neural excitability biases allocation and overlap of memory engrams. Journal of Neuroscience. 2024;44(21).

[18] Pikkarainen M, Rönkkö S, Savander V, Insausti R, Pitkänen A. Projections from the lateral, basal, and accessory basal nuclei of the amygdala to the hippocampal formation in rat. Journal of comparative neurology. 1999;403(2):229-60.

[19] Yoo S, Bae M, Tovar-Y-Romo L, Haughey N. Hippocampal encoding of interoceptive context during fear conditioning. Translational psychiatry. 2017;7(1):e991-1.

[20] Joshi SA, Aupperle RL, Khalsa SS. Interoception in fear learning and posttraumatic stress disorder. Focus. 2023;21(3):266-77.

[21] Talmi D. Enhanced emotional memory: Cognitive and neural mechanisms. Current Directions in Psychological Science. 2013;22(6):430-6.

[22] Girardeau G, Inema I, Buzsáki G. Reactivations of emotional memory in the hippocampus–amygdala system during sleep. Nature neuroscience. 2017;20(11):1634-42.

[23] Zaki Y, Pennington ZT, Morales-Rodriguez D, Bacon ME, Ko B, Francisco TR, et al. Offline ensemble co-reactivation links memories across days. Nature. 2025;637(8044):145-55.

[24] Popa D, Duvarci S, Popescu AT, Léna C, Paré D. Coherent amygdalocortical theta promotes fear memory consolidation during paradoxical sleep. Proceedings of the National Academy of Sciences. 2010;107(14):6516-9.

[25] Yang Y, Leopold DA, Duyn JH, Liu X. Hippocampal replay sequence governed by spontaneous brain-wide dynamics. PNAS nexus. 2024;3(4):pgae078.

[26] Cai DJ, Aharoni D, Shuman T, Shobe J, Biane J, Song W, et al. A shared neural ensemble links distinct contextual memories encoded close in time. Nature. 2016;534(7605):115-8.

[27] Choucry A, Nomoto M, Inokuchi K. Engram mechanisms of memory linking and identity. Nature Reviews Neuroscience. 2024:1-18.

[28] Martina M, Royer S, Paré D. Physiological properties of central medial and central lateral amygdala neurons. Journal of neurophysiology. 1999;82(4):1843-54.

[29] Monti JM, Monti D. Sleep disturbance in generalized anxiety disorder and its treatment. Sleep medicine reviews. 2000;4(3):263-76.

[30] Staner L. Sleep and anxiety disorders. Dialogues in clinical neuroscience. 2003;5(3):249-58.

[31] Pace-Schott EF, Germain A, Milad MR. Sleep and REM sleep disturbance in the pathophysiology of PTSD: the role of extinction memory. Biology of mood & anxiety disorders. 2015;5:1-19.

[32] van Liempt S. Sleep disturbances and PTSD: a perpetual circle? European journal of psychotraumatology. 2012;3(1):19142.

[33] Pace-Schott EF, Tracy LE, Rubin Z, Mollica AG, Ellenbogen JM, Bianchi MT, et al. Interactions of time of day and sleep with between-session habituation and extinction memory in young adult males. Experimental brain research. 2014;232:1443-58.

[34] Pace-Schott EF, Verga PW, Bennett TS, Spencer RM. Sleep promotes consolidation and generalization of extinction learning in simulated exposure therapy for spider fear. Journal of psychiatric research. 2012;46(8):1036-44.

[35] Jung T, Noh J. Alteration of fear behaviors in sleep-deprived adolescent rats: increased fear expression and delayed fear extinction. Animal Cells and Systems. 2021;25(2):83-92.

[36] Zhang J, Wang J, Wang Y, Zhang D, Li H, Lei Y. Sleep deprivation increases the generalization of perceptual and concept-based fear: An fNIRS study. Journal of Anxiety Disorders. 2024;105:102892.

[37] Zenses AK, Lenaert B, Peigneux P, Beckers T, Boddez Y. Sleep deprivation increases threat beliefs in human fear conditioning. Journal of sleep research. 2020;29(3):e12873.

[38] Foilb AR, Taylor-Yeremeeva EM, Schmidt BD, Ressler KJ, Carlezon Jr WA. Acute sleep deprivation reduces fear memories in male and female mice. bioRxiv. 2024.

[39] Cohen H, Ephraim-Oluwanuga OT, Akintunde OT, Gureje O, Matar MA, Todder D, et al. The potential beneficial effect of sleep deprivation following traumatic events to preventing PTSD: Review of current insight regarding sleep, memory, and trauma resonating with ancient rituals—Àìsùn Oku (African) and Tsuya (Japanese). Neuropsychopharmacology reports. 2023;43(1):2-11.

[40] Dudai Y, Karni A, Born J. The consolidation and transformation of memory. Neuron. 2015;88(1):20-32.

[41] Kunčická D, Krajčovič B, Stuchlík A, Brožka H. Neuroscientist's Behavioral Toolbox for Studying Episodic-Like Memory. eneuro. 2024;11(8).

[42] Kim WB, Cho JH. Encoding of contextual fear memory in hippocampal–amygdala circuit. Nature communications. 2020;11(1):1382.

[43] Lee JH, Kim WB, Park EH, Cho JH. Neocortical synaptic engrams for remote contextual memories. Nature Neuroscience. 2023;26(2):259-73.

[44] Preston AR, Eichenbaum H. Interplay of hippocampus and prefrontal cortex in memory. Current biology. 2013;23(17):R764-73.

[45] Matos MR, Visser E, Kramvis I, van der Loo RJ, Gebuis T, Zalm R, et al. Memory strength gates the involvement of a CREB-dependent cortical fear engram in remote memory. Nature Communications. 2019;10(1):2315.

[46] Restivo L, Vetere G, Bontempi B, Ammassari-Teule M. The formation of recent and remote memory is associated with time-dependent formation of dendritic spines in the hippocampus and anterior cingulate cortex. Journal of Neuroscience. 2009;29(25):8206-14.

[47] Norimoto H, Makino K, Gao M, Shikano Y, Okamoto K, Ishikawa T, et al. Hippocampal ripples down-regulate synapses. Science. 2018;359(6383):1524-7.

[48] Xia F, Richards BA, Tran MM, Josselyn SA, Takehara-Nishiuchi K, Frankland PW. Parvalbumin-positive interneurons mediate neocortical-hippocampal interactions that are necessary for memory consolidation. Elife. 2017;6:e27868.

[49] Ognjanovski N, Schaeffer S, Wu J, Mofakham S, Maruyama D, Zochowski M, et al. Parvalbumin-expressing interneurons coordinate hippocampal network dynamics required for memory consolidation. Nature communications. 2017;8(1):15039.

[50] Brewin CR, Dalgleish T, Joseph S. A dual representation theory of post-traumatic stress disorder. Psychological review. 1996;103(4):670.

[51] Bonsall MB, Holmes EA. Temporal dynamics of trauma memory persistence. Journal of the Royal Society Interface. 2023;20(203):20230108.

[52] Brewin CR, Burgess N. Contextualisation in the revised dual representation theory of PTSD: A response to Pearson and colleagues. Journal of behavior therapy and experimental psychiatry. 2014;45(1):217-9.

[53] Balooch SB, Neumann DL, Boschen MJ. Extinction treatment in multiple contexts attenuates ABC renewal in humans. Behaviour research and therapy. 2012;50(10):604-9.

[54] Hermann A, Stark R, Müller EA, Kruse O, Wolf OT, Merz CJ. Multiple extinction contexts modulate the neural correlates of context-dependent extinction learning and retrieval. Neurobiology of Learning and Memory. 2020;168:107150.

[55] Holmes NM, Westbrook RF. Extinction of reinstated or ABC renewed fear responses renders them resistant to subsequent ABA renewal. Journal of Experimental Psychology: Animal Behavior Processes. 2013;39(3):208.

[56] Neumann DL, Lipp OV, Cory SE. Conducting extinction in multiple contexts does not necessarily attenuate the renewal of shock expectancy in a fear-conditioning procedure with humans. Behaviour Research and Therapy. 2007;45(2):385-94.

[57] Bouton ME, García-Gutiérrez A, Zilski J, Moody EW. Extinction in multiple contexts does not necessarily make extinction less vulnerable to relapse. Behaviour research and therapy. 2006;44(7):983-94.

[58] Louie K, Wilson MA. Temporally structured replay of awake hippocampal ensemble activity during rapid eye movement sleep. Neuron. 2001;29(1):145-56.

[59] Brodt S, Inostroza M, Niethard N, Born J. Sleep—A brain-state serving systems memory consolidation. Neuron. 2023;111(7):1050-75.

[60] Girardeau G, Zugaro M. Hippocampal ripples and memory consolidation. Current opinion in neurobiology. 2011;21(3):452-9.

[61] Giri B, Miyawaki H, Mizuseki K, Cheng S, Diba K. Hippocampal reactivation extends for several hours following novel experience. Journal of Neuroscience. 2019;39(5):866-75.

[62] Tononi G, Cirelli C. Sleep function and synaptic homeostasis. Sleep medicine reviews. 2006;10(1):49-62.

[63] Tononi G, Cirelli C. Sleep and the price of plasticity: from synaptic and cellular homeostasis to memory consolidation and integration. Neuron. 2014;81(1):12-34.

[64] Miller KE, Brownlow JA, Gehrman PR. Sleep in PTSD: treatment approaches and outcomes. Current Opinion in Psychology. 2020;34:12-7.

[65] Colvonen PJ, Straus LD, Stepnowsky C, McCarthy MJ, Goldstein LA, Norman SB. Recent advancements in treating sleep disorders in co-occurring PTSD. Current psychiatry reports. 2018;20:1-13.

[66] Hutchison IC, Rathore S. The role of REM sleep theta activity in emotional memory. Frontiers in psychology. 2015;6:1439.

[67] Ravichandran NB, Lansner A, Herman P. Brain-like approaches to unsupervised learning of hidden representations-a comparative study. In: International Conference on Artificial Neural Networks. Springer; 2021. p. 162-73.

[68] Sandberg A, Lansner A, Petersson KM, Ekeberg. A Bayesian attractor network with incremental learning. Network: Computation in neural systems. 2002;13(2):179-94.

[69] Rao-Ruiz P, Yu J, Kushner SA, Josselyn SA. Neuronal competition: microcircuit mechanisms define the sparsity of the engram. Current opinion in neurobiology. 2019;54:163-70.

[70] Azizi AH, Wiskott L, Cheng S. A computational model for preplay in the hippocampus. Frontiers in computational neuroscience. 2013;7:161.

[71] Ji Z, Chu T, Dong X, Yu C, Bush D, Burgess N, et al. Dynamical Modulation of Hippocampal Replay Sequences through Firing Rate Adaptation. BioRxiv. 2024:2024-09.

[72] Pietras B, Schmutz V, Schwalger T. Mesoscopic description of hippocampal replay and metastability in spiking neural networks with short-term plasticity. PLOS Computational Biology. 2022;18(12):e1010809.

[73] Herry C, Ciocchi S, Senn V, Demmou L, Müller C, Lüthi A. Switching on and off fear by distinct neuronal circuits. Nature. 2008;454(7204):600-6.

[74] Kim J, Pignatelli M, Xu S, Itohara S, Tonegawa S. Antagonistic negative and positive neurons of the basolateral amygdala. Nature neuroscience. 2016;19(12):1636-46.

[75] An B, Kim J, Park K, Lee S, Song S, Choi S. Amount of fear extinction changes its underlying mechanisms. Elife. 2017;6:e25224.

[76] Myers KM, Ressler KJ, Davis M. Different mechanisms of fear extinction dependent on length of time since fear acquisition. Learning & memory. 2006;13(2):216-23.

[77] Cupolillo D, Grosjean N, Marneffe C, Viotti J, Reynaud C, Deforges S, et al. Early changes in the properties of CA3 engram cells explored with a novel viral tool. bioRxiv. 2024:2024-11.
