# Supplementary material for: Learning, sleep replay and consolidation of contextual fear memories: A neural network model
Source: PLoS Comput Biol. 2026 Mar 17;22(3):e1013251. doi: 10.1371/journal.pcbi.1013251 (PMC13012624; doi:10.1371/journal.pcbi.1013251)
Supplement: Attachment — Submitted filename: Reply_to_PLOS_Editor-1.pdf [file pcbi.1013251.s011.pdf]

Learning, sleep replay and consolidation of contextual fear memories: A neural network model – Reply to reviewers

Lars Werne, Angus Chadwick, Peggy Seriès

January 2026

We thank both reviewers 1 and 2 for their helpful replies throughout the review process. Below, we address the comments made at the latest stage.

Comments Reviewer 1

'Overall, the authors' responses are clear and thorough, and the revisions address most of the concerns. The architectural abstractions are now better motivated, the limitations of cortical memory decay are acknowledged, and parameter consistency across simulations is clarified. The discussion of valence-biased replay and memory linking is appropriate, even if these remain future extensions. All minor comments were handled cleanly. Together with the revisions made in response to Reviewer #2, the manuscript is substantially improved, and the authors' responses are generally satisfactory.'

We thank the reviewer for their positive comments, as well as for their kind assistance in improving the quality of our manuscript.

Comments Reviewer 2

'Thank you for the detailed responses to my review. I am satisfied with most of the authors' replies, particularly the inclusion of parameter sweeps, which strengthen the complex network model. Overall, I consider this work to be valuable and have recommended it for publication.'

We are grateful for the reviewer's guidance, assessment of our responses, and recommendation for publication.

'However, some of the network manipulations remain relatively coarse and lack detailed neurobiological grounding. As a result, there is still a gap between

the modelled phenomena and their biological interpretation, and alternative computational mechanisms might plausibly lead to similar results.'

It is true that our model operates at a fairly high level of abstraction, necessitated by its broad scope. Future modelling efforts will have the potential to focus more narrowly on any of the mechanisms posited by our model, in order to investigate them at a finer level of biological specificity. In the conclusion of our manuscript, we acknowledge that bridging the gap between our model's algorithms and their physical realization will be crucial for driving our understanding forward.

Below, we address the reviewer's additional suggestions.

R2-Min1

'For figures reporting results averaged over multiple simulation runs, measures of variability (e.g. s.e.m. or s.d.) should be included in the plots, for example in Fig. 4.'

We have added shaded bands to the curves in 4a, 4c, 5b and 9d, as a visual indicator for the standard deviation of the plotted quantities across runs (SD).

In Figure 4a, shaded regions denote the SD of the encoding strength of HIP, CTX, and BA_N engrams for a newly encountered context across runs. It is interesting to observe that *fast* synaptic changes – in HIP, or in BA_N following aversive inputs – result in a larger amount of variability across simulation runs.

In Figure 4c, the shaded regions represent the SD of the *percentage of contexts* (presented n days ago) that our model successfully recalled. The shaded bands appear relatively large; this observation does not indicate huge differences in recall performance across runs. Rather, it occurs because the percentage of recalled contexts from a given day equals either $0, 25, 50, 75$ or 100% on any run of the simulation. This discretization of the outcome measure makes large deviations from the mean quite likely on any run.

In Figure 5b, we evaluate our model's fear response when exposed to environments with a varying level of similarity to a context in which fear acquisition and extinction were performed. Shaded bands now indicate the SD of the model's percentage of active BA_P and BA_I cells, as well as of the fear response, across simulation runs.

Figure 9d *previously* illustrated the summed strength of all $BA_N \rightarrow BA_P$ synapses over the course of the 'delayed SEFL' simulation protocol – for model instances exposed to a 'traumatic stressor', as well as for a control group. This quantity *does* vary considerably across simulation runs, primarily because of the random sampling of (i) initial synapse weights, and of (ii) US signals presented

over the course of the simulation. To make the figure easier to interpret, and to limit the impact of the first source of variability on the plot, we have adjusted Figure 9d to instead show the models' *change* in the summed $BA_N \rightarrow BA_P$ synapse strength, relative to the onset of the traumatic stressor. Significant variability remains, but the model behaviour we intend to highlight can still be seen clearly: A drastic increase in BA synaptic density at the time of traumatic stress, continuing over the course of several *Perception-Sleep* cycles and persisting thereafter.

We insert the updated figures below. We have updated the figure captions in correspondence with these changes.

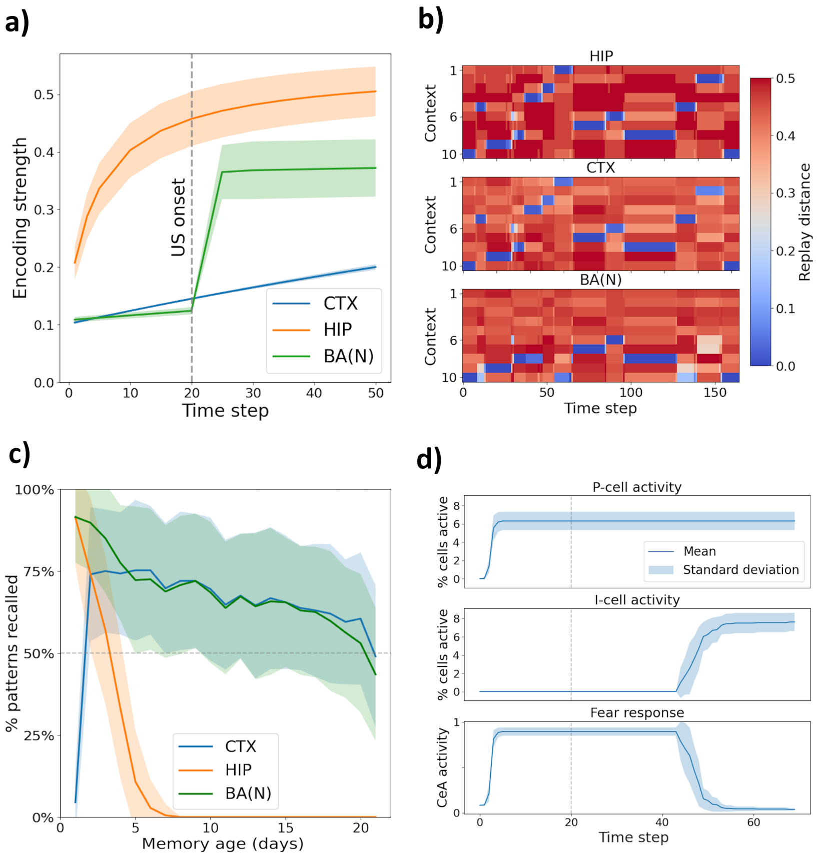

Figure 4: **Mechanisms of context/fear memory formation.**
We have added measures of across-run variability to panels a and c. Please see our revised manuscript for the updated caption.

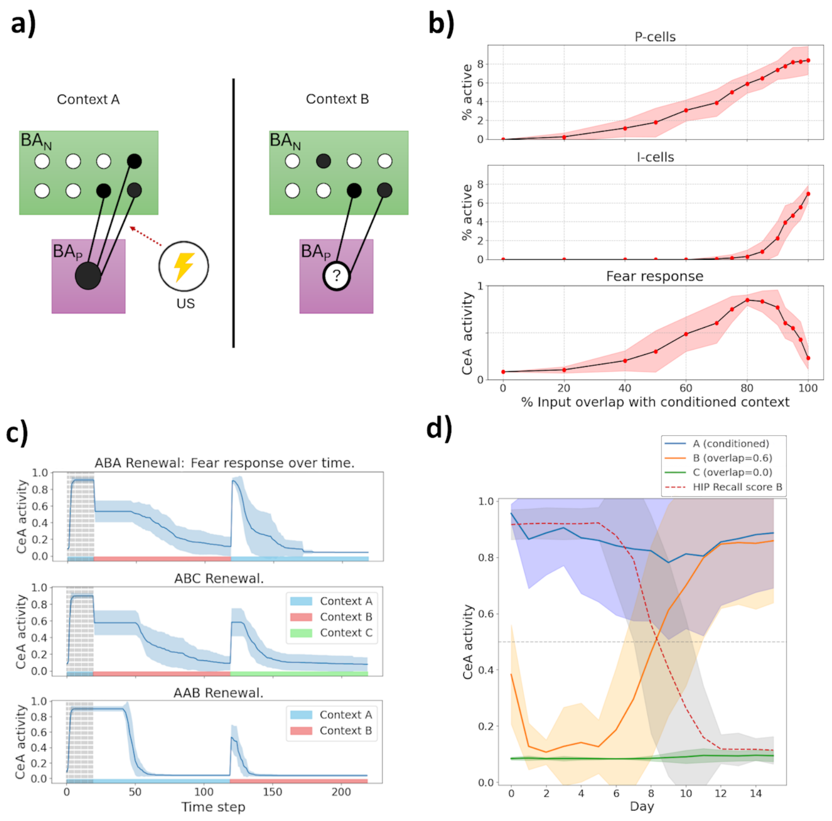

Figure 5: **Results on fear generalisation.**
We have added measures of across-run variability to panel b. Please see our revised manuscript for the updated caption.

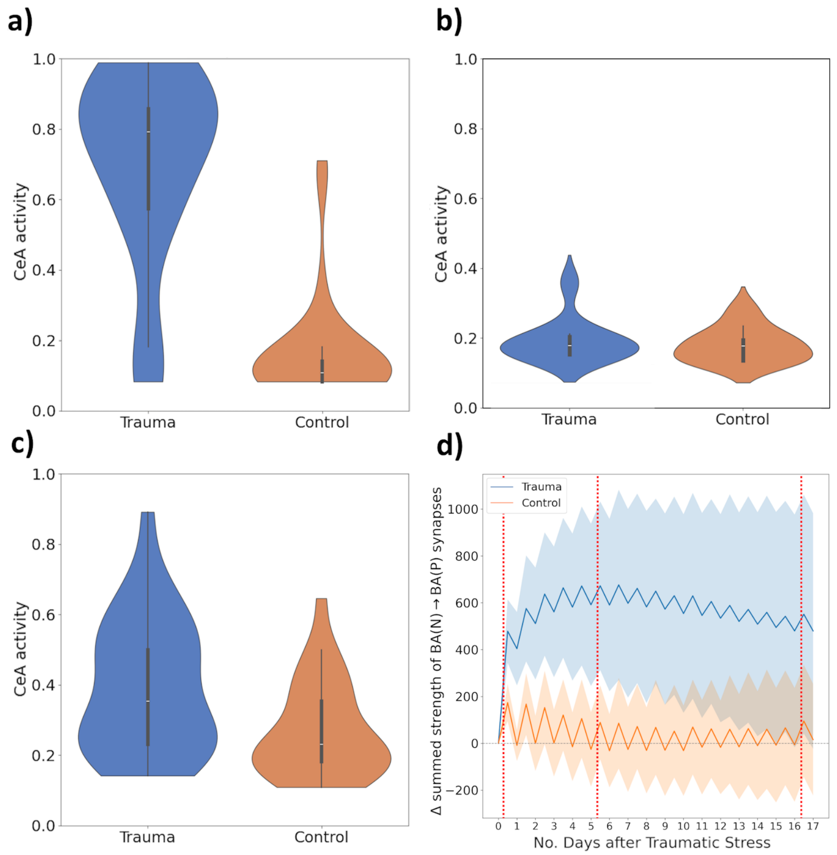

Figure 9: **Results on stress effects.**
We have added measures of across-run variability to panel d. Further, the panel now shows the *change* in the summed strength of the models' $BA_N \rightarrow BA_P$ synapses, rather than the total sum. Please see our revised manuscript for the updated caption.

R2-Min2

'Line 31: in PTSD — often emerge gradually, rather than immediately, after intense fearful or traumatic experiences. Appropriate citations are missing here.'

We thank the reviewer for correctly pointing this out. Previously, the sentence in question, discussing a limitation of existing computational models of fear learning, read as follows:

'As a result, they struggle to account for clinical observations where maladaptive fear symptoms — e.g., in PTSD — often emerge gradually, rather than immediately, after intense fearful, or traumatic, experiences.'

We have slightly revised this sentence, and have added appropriate citations:

'As a result, they struggle to account for gradual changes in fear recall over time (Poulos et al., 2016 [1]), which may be clinically relevant for the delayed onset or intensification of fear-related symptoms following trauma, as observed in delayed-onset PTSD (Utzon et al., 2014 [2]).'

The study by Poulos et al. reports incubation-like increases in fear recall in the days following intense fear conditioning with rats. Utzon et al. review the evidence on delayed-onset PTSD.

R2-Min3

'Line 50: citations 22–24 do not seem necessary in this context.'

We thank the reviewer for pointing this out and have removed the citations.

R2-Min4

'Line 90: Please consider adding a citation to the Hopfield (2010, PNAS) paper on mental exploration.'

We have now included the recommended citation in the following:

'Similar to past computational works on neural replay (Azizi et al., 2013 [3]; Ji et al., 2024 [4]; Hopfield, 2010 [5]), HIP in our model thus relies on a combination of excitatory attractor dynamics and a self-inhibitory fatigue mechanism to cycle through different memory patterns (Pietras et al., 2022 [6]).'

References

[1] Poulos AM, Mehta N, Lu B, Amir D, Livingston B, Santarelli A, et al. Conditioning-and time-dependent increases in context fear and generalization. Learning & Memory. 2016;23(7):379-85.

[2] Utzon-Frank N, Breinegaard N, Bertelsen M, Borritz M, Eller NH, Nordentott M, et al. Occurrence of delayed-onset post-traumatic stress disorder: a systematic review and meta-analysis of prospective studies. Scandinavian journal of work, environment & health. 2014:215-29.

[3] Azizi AH, Wiskott L, Cheng S. A computational model for preplay in the hippocampus. Frontiers in computational neuroscience. 2013;7:161.

[4] Ji Z, Chu T, Dong X, Yu C, Bush D, Burgess N, et al. Dynamical Modulation of Hippocampal Replay Sequences through Firing Rate Adaptation. BioRxiv. 2024:2024-09.

[5] Hopfield JJ. Neurodynamics of mental exploration. Proceedings of the National Academy of Sciences. 2010;107(4):1648-53.

[6] Pietras B, Schmutz V, Schwalger T. Mesoscopic description of hippocampal replay and metastability in spiking neural networks with short-term plasticity. PLOS Computational Biology. 2022;18(12):e1010809.
